# Conventional, High-Resolution and Imaging Flow Cytometry: Benchmarking Performance in Characterisation of Extracellular Vesicles

**DOI:** 10.3390/biomedicines9020124

**Published:** 2021-01-27

**Authors:** Jaco Botha, Haley R. Pugsley, Aase Handberg

**Affiliations:** 1 Department of Clinical Biochemistry, Aalborg University Hospital, North Denmark Region, DK-9000 Aalborg, Denmark; aaha@rn.dk; 2 Department of Clinical Medicine, Aalborg University, DK-9000 Aalborg, Denmark; 3 Luminex Corporation, Seattle, WA 98119, USA; hpugsley@luminexcorp.com

**Keywords:** extracellular vesicles, exosomes, microvesicles, flow cytometry, imaging flow cytometry, high-resolution flow cytometry, submicron particle analysis, standardisation, repeatability, reproducibility

## Abstract

Flow cytometry remains a commonly used methodology due to its ability to characterise multiple parameters on single particles in a high-throughput manner. In order to address limitations with lacking sensitivity of conventional flow cytometry to characterise extracellular vesicles (EVs), novel, highly sensitive platforms, such as high-resolution and imaging flow cytometers, have been developed. We provided comparative benchmarks of a conventional FACS Aria III, a high-resolution Apogee A60 Micro-PLUS and the ImageStream X Mk II imaging flow cytometry platform. Nanospheres were used to systematically characterise the abilities of each platform to detect and quantify populations with different sizes, refractive indices and fluorescence properties, and the repeatability in concentration determinations was reported for each population. We evaluated the ability of the three platforms to detect different EV phenotypes in blood plasma and the intra-day, inter-day and global variabilities in determining EV concentrations. By applying this or similar methodology to characterise methods, researchers would be able to make informed decisions on choice of platforms and thereby be able to match suitable flow cytometry platforms with projects based on the needs of each individual project. This would greatly contribute to improving the robustness and reproducibility of EV studies.

## 1. Introduction

Over the years, several methodological platforms have been developed and used to characterise extracellular vesicles (EVs) in biological samples. Two common methodologies used to characterise the size and concentration of EVs in samples are nanoparticle tracking analysis (NTA) [1] and tuneable resistance pulse sensing (TRPS) [2]. While capable of detecting particles in size range of EVs, NTA and TRPS lack the ability to discriminate between EVs and other particles with similar physical properties, such as lipoproteins, co-isolated protein aggregates and label aggregates or micelles [3,4,5,6,7]. Therefore, it has been recommended that they be supplemented with other methods to confirm the presence of proteins associated with the EV proteome in the sample, such as Western blot, ELISA or bead-based flow cytometric characterisation [8,9,10]. However, these methods only provide information on the content of specific EV markers in the bulk of the sample or EV isolates and are unable to distinguish between variations in the number of EVs or differences in the expression of the specific marker, and, at best, they only provide tentative evidence that the marker of interest is present on EVs. Therefore, it has become common practice to further supplement these methods with transmission electron microscopy (TEM) to confirm the presence of these markers on the surface of EVs, determine EV sizes and physical properties, to some extent, and the presence of other contaminants in the sample [11,12,13]. Thus, the whole process becomes quite labour intensive, expensive and time consuming while remaining incapable of multi-parametric characterisation of single particles.

As such, flow cytometry has remained a popular method for the characterisation of EVs since it provides the ability to characterise multiple parameters on single particles in a high-throughput manner while providing some information regarding their size based on the relative amount of scattered light. However, it has been well documented that most conventional flow cytometers have a lower detection limit between 300 nm and 500 nm and a best-case minimal detection limit of about 150 nm by using optimised triggering strategies, thereby excluding the abundantly present smaller EVs from the analysis [14,15,16]. Another significant limitation with conventional flow cytometers is the detection of multiple EVs as a single event—so-called coincident or “swarm” detection—which is due to the fluidics being optimised for characterisation of particles in the size range of cells (2–30 µm) [17,18,19]. Therefore, due to only being able to detect a small proportion of EVs and exosomes regardless of characterisation strategy and the possibility of detecting multiple EVs as a single event, the applicability of conventional flow cytometers in the field of EV research has been widely disputed.

In order to address the limitations of conventional flow cytometers in EV characterisation, several attempts have been made to increase their sensitivity towards these small particles, thereby giving rise to high-resolution flow cytometers (hFCM). Common modifications include changing photodiodes on light scatter channels to photomultiplier tubes (PMTs), the addition of a reduced wide-angle forward scatter/medium-angle light scatter (MALS) collection, installation of lasers with higher power and decreases in sample and sheath flow rates [20,21,22,23]. With these modifications, high-resolution flow cytometers have been reported to have a lower detection limit below that of conventional flow cytometers based on the detection of synthetic nanospheres, and their use is becoming increasingly prevalent in the field of EV research [14,20,22,23,24].

Advanced imaging flow cytometry has previously been shown to have a significant advantage towards detection and characterisation of particles in the submicron range by being able to detect synthetic nanospheres with sizes down to 20 nm [25]. Much of this increased sensitivity is owed to the usage of charge-coupled device (CCD) cameras, which have a much larger dynamic range and lower noise than PMTs. Another significant factor that improves the ability of imaging flow cytometers to detect dim signals is time delay integration (TDI) of pixel intensities of each particle as it flows past the CCD cameras. Along with slow sheath and sample flow rates, this allows for longer signal integration times for each particle, thus leading to increased sensitivity. Additionally, unlike traditional flow cytometry, images of all particles acquired are saved, thereby allowing for far more powerful data analysis and event classification. As such, imaging flow cytometry has been demonstrated to be an efficient tool for characterising EVs [26,27,28,29].

Even though these and other platforms have been available for some time, few attempts have been made to benchmark different platforms in a fair comparative manner. Thus, the aim of this study was to provide a benchmark of a conventional flow cytometer, a high-resolution flow cytometer and an imaging flow cytometer. First, we assessed the ability of each platform to detect, resolve and quantify particles within the EV size range. Next, we evaluated their ability to detect different EV phenotypes directly in a complex biological fluid using blood plasma as a surrogate. Finally, intra-day, inter-day and global variability in detecting different EV phenotypes in blood plasma were determined for each platform.

## 2. Materials and Methods

### 2.1. Collection of Platelet-Poor Blood Plasma

Blood for this study was collected from anonymous healthy Danish blood donors and kindly donated by the Danish Blood Bank (Department of Clinical Immunology, Aalborg University Hospital, Aalborg, Denmark) in accordance with local ethical regulations regarding health scientific research on anonymous human biological material (*Komitéloven §14*, *stk. 3*) and the Helsinki Declaration. Waste blood used to prime the tube prior to blood donation was collected into BD Vacutainer™ 9NC tubes containing a final concentration of 0.0105M NA_3_ Citrate (BD Biosciences, San Jose, CA, USA, Cat. no. 366075), and platelet-poor plasma (PPP) was acquired by a two-step centrifugation procedure initiated within an hour after collection, as previously described [30]. First, whole blood was centrifuged at 2500× *g* for 15 min at room temperature, after which platelet-rich plasma was collected > 1 cm above the buffy coat and transferred into a new tube. Second, platelet-rich plasma was subjected to additional centrifugation at 2500× *g* for 15 min at room temperature, and PPP was collected > 1 cm above the pellet and transferred into a container, where all samples were pooled. Finally, the PPP pool was thoroughly mixed and divided into 0.5 mL aliquots in 1.5 mL tubes and stored at −80 °C until use.

### 2.2. Staining of Platelet-Poor Plasma

Prior to labelling, PPP was thawed at room temperature. Thawed PPP was centrifuged at 1850× *g* for 5 min to remove potential clots that formed during freezing/thawing, and the supernatant was transferred to a separate tube, from which it was aliquoted. Antibodies were centrifuged at 17,000× *g* for 10 min prior to staining, and a master mix was prepared of both specific and matched isotype control antibodies, as described in Table 1. In brief, fluorescein isothiocyanate (FITC)-conjugated Lactadherin, a protein molecule that binds to the lipid phosphatidylserine in a stereospecific and calcium-independent manner [31], was used in both the specific and isotype control master mixes to detect phosphatidylserine-exposing EVs. In the specific antibody master mix, allophycocyanin (APC)-conjugated anti-CD41 antibody against the platelet surface marker CD41 abundantly expressed on platelet EVs [32] and phycoerythrin (PE)-conjugated anti-CD36 antibody against the class B lipid scavenger receptor CD36 present on most cells [33] were used to identify EV populations that could be discriminated easily and less easily from the background, respectively. In the isotype control master mix, specific isotype control antibodies were used for anti-CD41 and anti-CD36 matched on the organism, immunoglobulin class (heavy chain and light chain), clonality and fluorophore:protein ratio. Fifty microliters of PPP was labelled directly with 25 µL master mix for 30 min on ice in the dark. Unlabelled controls were prepared by adding 25 µL buffer to 50 µL PPP, and buffer controls were prepared by adding 25 µL master mix to 50 µL buffer and incubating in the same manner as samples. After incubation, the reaction was halted by diluting stained samples and controls with Dulbecco’s phosphate-buffered saline (DPBS) to a final volume of 850 µL (final dilution: 17-fold). Preparations were kept on ice in the dark until analysis. In addition to the above-mentioned controls, a 1% Triton X-100 detergent lysis control was prepared by transferring 180 µL of stained samples into a pre-chilled tube containing 20 µL 10% Triton X-100. The detergent lysis control was vortexed for 30 s, incubated for at least 30 min on ice and again vortexed for 30 s immediately prior to analysis. Finally, single-stained compensation controls were prepared for each analysis day and platformed for FITC, PE, and APC.

### 2.3. BD FACSAria™ III

Conventional flow cytometry was performed on a BD FACSAria™ III High-Speed Cell Sorter (BD Biosciences, San Jose, CA, USA) equipped with 375 nm (7 mW), 488 nm (20 mW) and 633 nm (18 mW) lasers. The 488 nm and 633 nm lasers were run at maximum power, while the 405 nm laser was turned off and not utilised in this study. Forward scatter (FSC) and side scatter (SSC) were collected from the 488 nm laser into a photodiode and a PMT, respectively. FITC was collected from the 488 nm laser through a 530/30 bandpass filter. PE was collected from the 488 nm laser through a 585/42 bandpass filter. APC was collected from the 633 nm laser through a 660/20 bandpass filter. Data were acquired for 180 s at 70 psi and a sample flow rate set to 2 to allow for a narrow sample core while maintaining an adequate event rate. Nanosphere samples were either acquired with a triggering threshold set to 200 on side scatter or FITC. All other samples were acquired with a triggering threshold set to 200 on FITC. Triggering thresholds were set above electronic noise, while PMT voltages on forward and side scatter were adjusted to give the optimal separation between background and the smallest discriminable nanosphere population in the mixture described below. Similarly, the triggering threshold was set to 200 on FITC, and the PMT voltage was adjusted to give the optimal separation between background and fluorescent nanospheres in the same mixture. Instrument settings were adjusted, and the data were recorded in BD FACSDiva Software v. 8.0.2 (BD Biosciences). All data from the FACS Aria III was acquired in the FCS v. 3.1 format and processed in FlowJo v. 10.5.3 (FLowJo LLC, BD Biosciences, San Jose, CA, USA).

### 2.4. Apogee A60 Micro-PLUS

High-resolution flow cytometry was performed on an A60 Micro-PLUS flow cytometer (Apogee Flow Systems, Hemel Hempstead, UK) equipped with 405 nm (300 mW), 647 nm (180 mW) and 488 nm (200 mW) diode lasers. The 405 nm laser was run at 190 mW, while the 488 nm and 638 nm lasers were run at 100 mW. Three PMTs were factory-fitted for the collection of small-angle light scatter (SALS), medium-angle light scatter (MALS) and large-angle light scatter (LALS) signals collected from the 405 nm laser after being separated from fluorescence signals on this laser by an LP415 long-pass filter. FITC signals were collected from the 488 nm laser into a PMT fitted with a 530/40 bandpass filter, PE signals were collected from the 488 nm laser into a PMT with a 575/30 bandpass filter, and APC signals were collected from the 638 nm laser into a PMT with a 680/35 bandpass filter. Data were acquired for 180 s at a sample flow rate of 0.75 µL/min and a sheath fluid pressure of 150 mBar in order to keep the sample core as tight as possible and allow for adequate exposure of EVs by the lasers. Nanosphere samples were acquired with a triggering threshold set to 30 on MALS corresponding to an intensity value of 1920 on this parameter. Plasma samples were acquired with a triggering threshold set to 30 on MALS and 20 on FITC, which correspond to values of 1920 and 1280 on these channels, respectively. The triggering threshold on MALS was set to allow the collection of 100 events/second in unlabelled PBS, whereas the triggering threshold on FITC was set to allow the acquisition of 100 events/second in unlabelled PPP. Instrument settings were adjusted, and the data were recorded in the Histogram v. 1.21 software utility (Apogee Flow Systems, Hemel Hempstead, Hertfordshire, UK). All data from the A60 Micro-PLUS were acquired in the FCS v. 3.0 format and analysed in FlowJo.

### 2.5. Amnis^®^ ImageStream^®^ X Mk II

Imaging flow cytometry was performed on an ImageStream X Mk II imaging flow cytometer (Luminex Corporation, Seattle, WA, USA). The ImageStream X Mk II was equipped with 488 nm (200 mW), 642 nm (150 mW), 405 nm (120 mW), 785 nm (70 mW), 561 nm (200 mW) and 592 nm (300 mW) lasers. All lasers were run at their maximum power with the exception of the 561 nm and 592 nm lasers, which were turned off completely and not utilised in this study. All signals were collected into 12 channels distributed on two charge-coupled devices (CCDs) with 6 channels per camera with a filter setup. The notch filter on the 405 nm laser containing a 435 nm longpass filter was removed from the 405 nm laser in order to enable acquisition of side scatter from this laser in channel 7 (Ch07) in addition to side scatter from the 785 nm laser, which was collected into channel 6 (Ch06). FITC signals were collected from the 488 nm laser into channel 2 (Ch02) on camera 1 through a 528/65 bandpass filter. PE was collected from the 488 nm laser into channel 3 (Ch03) on camera 1 through a 577/35 bandpass filter. APC signals were collected from the 642 nm laser into channel 11 (Ch11) on camera 2 through a 702/85 bandpass filter. One channel on each CCD (channel 1 and channel 9; Ch01 and Ch09, respectively) was reserved for the bright field (BF) and used for cross camera alignment of the two CCDs. Data acquisition was disabled for all other channels than the above-mentioned. Speed beads (1.0 ± 0.2 µm COOH-functionalised polystyrene beads, Refractive Index (RI) = 1.63) were run constantly during sample acquisition and used for sample core alignment, focussing and camera synchronisation. Data were acquired for 180 s at 60× magnification with a numerical aperture of 0.9 and image resolution of 0.3 × 0.3 µm/pixel with extended depth of field (EDF) turned off. The diameter of the sample core was adjusted to and kept at 6 µm to keep most particles in focus while maintaining reasonable sample core stability. Data acquisition was initialized approximately three minutes after loading each sample, as this was the time required for event rates to stabilise due to dilution of the sample with sheath fluid. Instrument settings were adjusted, and the data were recorded in the INSPIRE™ v200.1.388 software utility (Luminex Corporation, Seattle, WA, USA). Data were saved as raw image files (RIF) after the acquisition. After compensation and data analysis, compensated (CIF) and data analysis file (DAF) were created for each file. Data from nanosphere mixtures were first compensated for fluorescence spill-over in IDEAS^®^ v. 6.2, and CIF/DAF files exported as FCS files, which were then analysed in FlowJo v. 10.5.3. All other analyses were performed in IDEAS. Initially, the standard “Mask Combined” (MC) masking settings were used, which is based on Boolean OR logic, where any signal in any of the acquired channels contributes to the constructed mask. However, due to most small nanospheres and EVs not having a visible signal in the bright field channel, these channels were found to create artefacts, which resulted in erroneous quantification in other channels. Thus, a new mask was constructed for all other channel masks (“M + channel number”) in which bright field masks were not equated (“M02 OR M03 OR M06 OR M07 OR M11”) and used instead.

### 2.6. Detection and Quantification of Silica and Polystyrene Nanospheres

In order to compare the ability of the different platforms to measure particles in the EV size, light scatter and fluorescence range, a mixture of non-fluorescent silica and fluorescent polystyrene nanospheres was used. The mixture consisted of 850 µL Apogee Mix (Apogee Flow Systems; Cat. no. 1493; Lot. no. CAL0098) spiked with 200 times pre-diluted 280 nm multi-fluorescent Ultra Rainbow Beads (Spherotech Inc., Lake Forest, IL, USA; Cat. no. URFP-02-2), thereby yielding a mixture with the following populations: 180 nm, 240 nm, 300 nm, 590 nm, 880 nm and 1300 nm non-fluorescent silica nanospheres (RI = 1.47) and 110 nm yellow-green fluorescent, 280 nm multi-fluorescent and 500 nm yellow-green fluorescent polystyrene nanospheres (RI = 1.63). For platforms capable of detecting the 180 nm non-fluorescent silica nanosphere population, in-house 100 nm yellow-green silica nanospheres were additionally analysed in order to investigate their ability to discriminate particles with very little light scatter from the background. Further details on data acquisition and analysis can be found in the appropriate sections of methods for each platform, results and figure legends.

### 2.7. Flow Cytometric Characterisation of EVs

**Figure 1:**
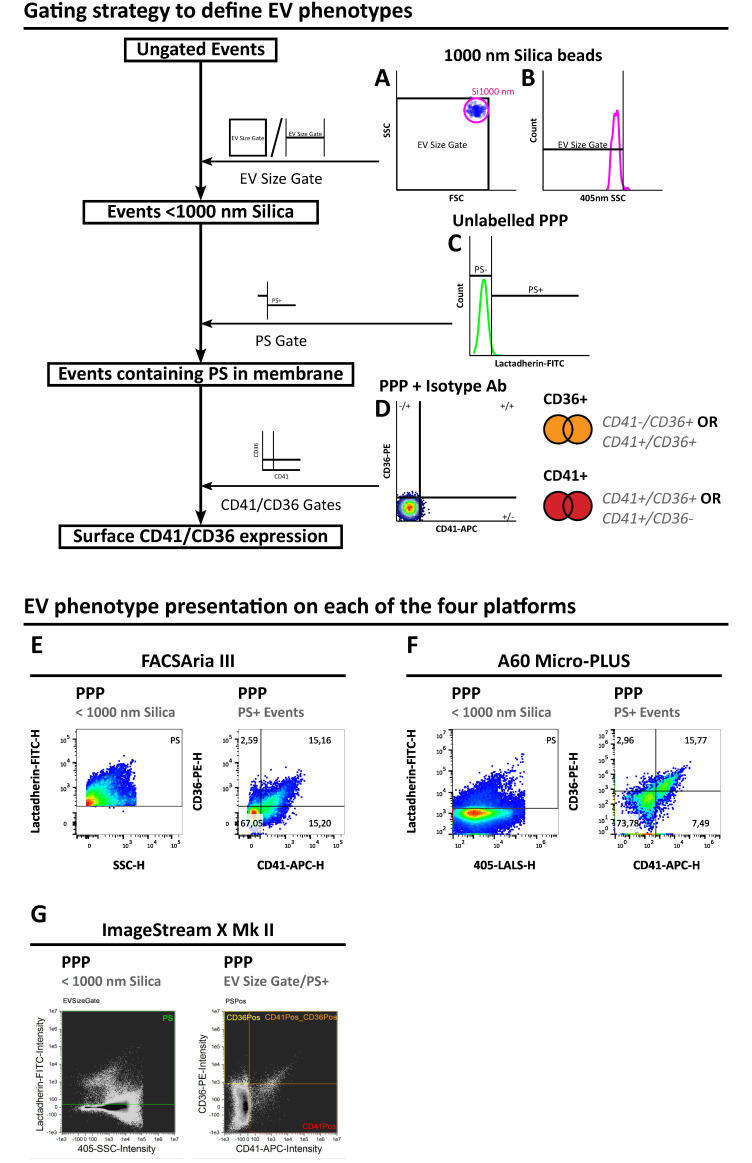
EV-defining gating strategy and depiction of representative data for each of the three flow cytometry platforms. (**A**–**D**) Schematic depiction of the gating strategy used for defining EV phenotypes in PPP samples. (**A**) First, a size gate was established on the 99th percentile of green fluorescent 1000 nm silica nanospheres (RI 1.42) on FSC/SALS vs. SSC/LALS on FACS Aria III and Apogee A60 Micro-PLUS or (**B**) on 405 nm SSC alone on ImageStream X Mk II. (**C**) Next, on events within the EV size gate in an unlabelled PPP sample, a gate was established on the 99th percentile in the FITC channel to define lactadherin-binding phosphatidylserine (PS)+ events (Apogee A60 Micro-PLUS and ImageStream X Mk II) or set to the triggering threshold value of 200 (FACS Aria III). (**D**) On PS+ events in a PPP sample stained with isotype control antibodies, quadrant gates were established on the 99th percentiles of events in the APC vs PE channels to define different combinations of CD41+/− and CD36+/− events, respectively. (**D**) Finally, the sums of CD41+ and CD36+ events were defined by establishing logical OR gates on “CD41+|CD36+ OR CD41+|CD36−“ and “CD41−|CD36+ OR CD41+|CD36+”, respectively. (**E**–**G**) Representative histograms of PS+ events (left) or scatterplots of CD41+/−|CD36+/− events (right) for (**E**) FACS Aria III, (**F**) Apogee A60 Micro-PLUS and (**G**) ImageStream X Mk II. EV: Extracellular vesicle; PPP: Platelet poor plasma; PS: Phosphatidylserine; FITC: Fluorescein isothiocyanate; APC: Allophycocyanin; PE: Phycoerythrin; SSC: Side scatter; LALS: Large angle light scatter.

The general gating strategy for defining the different EV phenotypes on all platforms in this study is depicted in Figure 1, with platform-specific strategies depicted in supplementary Figures S1–S3. First, a <1000 nm size gate was established on a sample of 1000 nm yellow-green fluorescent silica nanospheres (RI = 1.47), encompassing all values below the 99th percentile of the 1000 nm bead population on forward scatter and side scatter (FACS Aria III and A60 Micro-PLUS; Figure 1A) or 405 nm side scatter alone (ImageStream X Mk II; Figure 1B). Next, a phosphatidylserine (PS) expression gate (Figure 1C) was established on all events < 1000 nm silica in the lactadherin-FITC channel on an unlabelled PPP sample (A60 Micro-PLUS and ImageStream X Mk II) or set to the triggering threshold value on the FITC channel (FACS Aria III). Phenotype gates were then defined on bi-variate plots of PS-expressing events in a PPP sample stained with isotype control antibodies of CD36-PE and CD41-APC channels using quadrant gates on the 99th percentiles of both parameters to define events positive for the expression of these markers (Figure 1D). Finally, Boolean logic gates were used to define the total number of CD36+ and CD41+ events (FACS Aria III and A60 Micro-PLUS). On the ImageStream X Mk II, however, PS+|CD41+, PS+|CD36+ and PS+|CD41+|CD36+ different phenotypes were defined separately with square gates instead on a bivariate plot of CD41 and CD36 expression (Figure 1G and Figure S3). Thus, four phenotypes were defined as being of interest in this benchmarking study: the EV-defining PS+ phenotype; an easily discriminable PS+|CD41+ phenotype; a less discriminable PS+|CD36+ phenotype; a triple-stained PS+|CD41+|CD36+ phenotype. These phenotypes are depicted in Figure 1E–G.

In order to assess whether true EV phenotypes were being measured, different controls were employed to account for common artefacts and pitfalls. Unlabelled samples were used to establish the EV-defining gate of this study, thereby excluding a large proportion of non-EV particles from the analysis. Similarly, gating on well-defined isotype controls limited the inclusion of events resulting from non-specific binding of antibodies and fluorescence background caused by the unbound stain. Buffer controls stained with either specific or isotype control master mixes were analysed in order to account for the presence of label aggregates in samples, which could confound EV concentrations and confer variability across analysis days. Detergent lysis controls were analysed in order to assess whether the measured phenotypes contained membrane-like structures, which are disrupted when detergent is added. Population distributions and concentrations of all phenotypes were comparatively analysed between samples labelled with specific antibodies and controls. For each platform, a serial dilution control of the sample was prepared to ensure detection of single events, where event rate linearity and median fluorescence signal intensity stability were evaluated within this range (dilution factors: 5, 9, 17, 33 and 65-fold). Controls are depicted in supplementary results (Figures S4–S7).

Gates were constructed for each analysis day and transferred to all samples and controls from the same analysis day. Concentrations were calculated as the number of events divided by the sample volume and multiplied by the dilution factor. For the FACS Aria, however, BD TruCount™ counting beads (BD Biosciences, Cat. no. 340334) were utilised to account for the instability of the sample flow rate. Here, the concentrations were calculated based on the number of measured counting beads, as specified by the manufacturer in the technical datasheet. All statistics were batch exported as either .xls files (FACS Aria III, A60 Micro-PLUS) or .CSV files (ImageStream X Mk II) for further analysis.

### 2.8. Data Analysis

Data processing and statistical data analysis were performed in R v. 3.5.1 (R Core Team, Vienna, Austria) in RStudio v. 1.1.456 using the xlsx [34], ggplot2 [35], reshape2 [36] and grid [37] packages installed. The assumption of normality was tested with Shapiro–Wilk’s W-test for normality and confirmed with QQ-plots and histograms of distributions. Homoscedasticity was assessed with Levene’s test for equal distributions and confirmed visually with different plots. Due to either non-normal distributions or heteroscedasticity between groups, the unpaired Wilcoxon Rank Sums test was used to compare parameters between groups. *p*-values were adjusted with Holm’s sequentially rejective multiple test procedure [38]. All *p*-values were reported as two-sided, and statistical significance was defined as *p* < 0.05.

## 3. Results

### 3.1. Detection and Quantification of Synthetic Nanospheres with Different Sizes and Refractive Indices

In order to characterise the bulk of EVs effectively, instruments must be able to reliably detect, quantify and gather light scatter and fluorescence signals from single EVs. Thus, we decided to compare the abilities of the four platforms to detect and quantify particles in size, scatter and fluorescence range of EVs. To this end, we analysed a single replicate of a mixture of synthetic nanospheres consisting of nine different nanosphere populations with defined sizes, refractive indices and fluorescence properties (non-fluorescent silica nanospheres: 180 nm, 240 nm, 300 nm, 590 nm, 880 nm, 1300 nm; fluorescent polystyrene nanospheres: 110 nm yellow-green fluorescent, 280 nm multi-fluorescent, 500 nm yellow-green fluorescent). Additionally, a monodisperse solution of 100 nm dimly yellow-green fluorescent silica nanospheres was analysed on platforms capable of discriminating 180 nm silica beads from the background on each analysis day to assess their sensitivity in detecting such small particles.

Previous studies have mainly focussed on the ability of flow cytometers to detect and discriminate particles of different sizes from each other on single light scatter parameters when determining instrument sensitivity [24,25,29,39]. We, however, decided against this approach, as discrimination of particles with different sizes in this manner is highly dependent on collection angles, light sources and optics utilised in the system [40], and better results can often be obtained by using more than one parameter when gating populations in samples. Instead, we determined the limits of detection (LoD) and limits of quantification (LoQ) for each platform and compared their abilities to quantify each individual nanosphere population.

#### 3.1.1. Limits of Detection and Quantification

In this study, LoD was defined as the smallest nanosphere population, which could be confidently identified and discriminated from the background and other nanosphere populations. LoQ was defined as the smallest bead population, which could be quantified with sufficient reproducibility. In terms of reliability, we decided on an arbitrary accuracy goal of coefficient of variability (CV) less than 20%. In a sense, it can be assumed that LoD ≤ LoQ. In order to maximise instrument performance, we decided to use optimal instrument settings and gating strategies for each platform.

**Figure 2:**
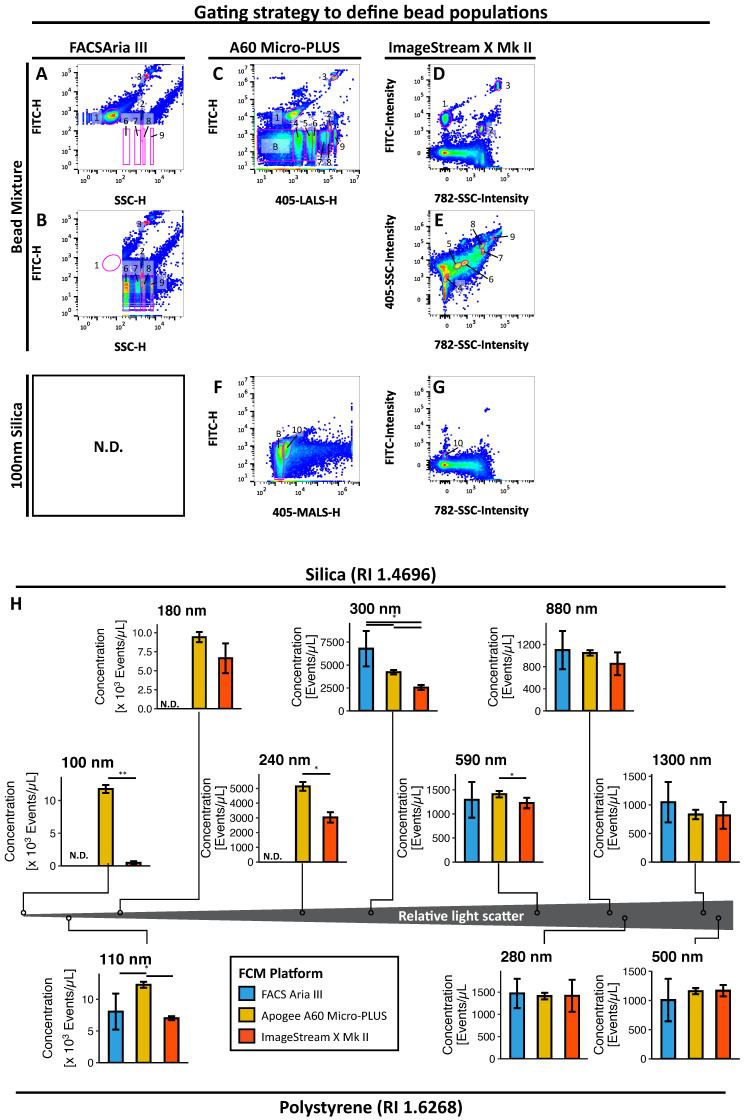
Detection and quantification of nanospheres with different refractive indices. (**A**–**G**) Gating strategy to define each nanosphere population in the bead mixture on (**A**) FACS Aria III for triggering in the FITC and (**B**) SSC, (**C**) Apogee A60 Micro-PLUS and (**D**,**E**) ImageStream X Mk II. (**F**,**G**) Gating strategy to define dimly FITC fluorescent 100 nm silica nanospheres on (**F**) Apogee A60 Micro-PLUS and (**G**) ImageStream X Mk II. (**H**) Concentrations of silica (top) and fluorescent polystyrene (bottom) nanosphere populations measured by each of the four platforms with an indication to their relative light scatter. Results are depicted as mean (bar) ± 1SD (error bars) for *n* = 5 observations of each nanosphere population acquired over the course of five analysis days. Numbers in A-E represent different nanosphere populations as follows: 1: Ps110 nm; 2: Ps280 nm; 3: Ps580 nm; 4: Si180 nm; 5: Si240 nm; 6: Si300 nm; 7: Si590 nm; 8: Si880 nm; 9: Si1300 nm; 10: Si100 nm; B: Background. *: *p* < 0.05; **: *p* < 0.01. N.D.: Not detected; RI: Refractive Index; MALS: Medium angle light scatter.

**Figure 3:**
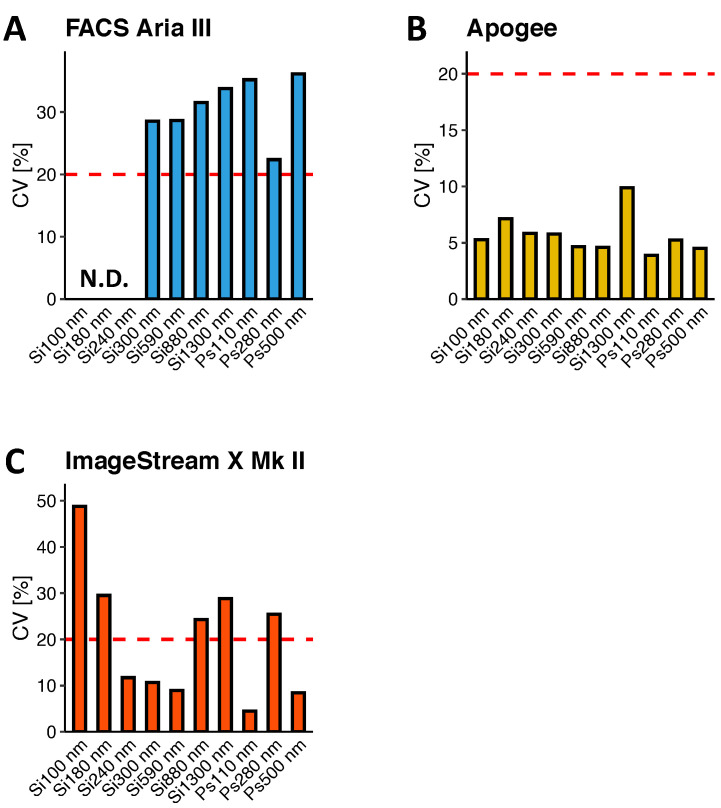
Variability in concentration determinations of synthetic nanospheres on (**A**) FACS Aria III, (**B**) Apogee A60 Micro-PLUS and (**C**) ImageStream X Mk II. The horizontal red, dashed line represents the arbitrary limit of quantification (LoQ) set in this study to a coefficient of variability (CV) of less than 20% for concentrations of synthetic nanospheres. CV: Coefficient of variability = standard deviation (SD)/mean for *n* = 5 per nanosphere population per platform; N.D.: Not detected.

By using a triggering threshold set to 200 on SSC, FACS Aria III was unable to detect and resolve silica nanosphere populations below 300 nm or the 110 nm fluorescent polystyrene nanospheres (Figure 2A). When using a triggering threshold set to 200 on the green channel on the 488 nm laser (FITC), as previously described by Arraud et al. [15,16], FACS Aria III could detect 110 nm fluorescent polystyrene nanospheres with light scatter below the 300 nm silica nanospheres. However, this was at the cost of the non-fluorescent silica nanosphere populations and the dimly fluorescent 280 nm polystyrene nanospheres, where the latter could not be resolved from non-fluorescent populations regardless of triggering threshold (Figure 2B). Although able to detect different nanospheres, FACS Aria III could not quantify any of these with a sufficient reproducibility (Figure 3A, CV = 22.4–36.1%).

Apogee A60 Micro-PLUS was able to detect all nanosphere populations in the nanosphere mixture with a triggering threshold set on MALS to filter out noise, and all nanosphere populations could be resolved from the background on LALS. In addition, all fluorescent polystyrene nanosphere populations could be resolved from the background based on their fluorescence; however, the 280 nm nanospheres were only barely resolvable from the background based on their fluorescence (Figure 2C). This setting also allowed for the detection and identification of 100 nm dimly fluorescent silica nanospheres on a biaxial plot of MALS and FITC, although these nanospheres could not be resolved completely from the background based on light scatter, and they significantly overlapped background with regards to fluorescence (Figure 2G). Moreover, all silica and polystyrene nanosphere populations could be quantified with sufficient reproducibility (Figure 3B, CV = 3.9–9.9%).

Although ImageStream X Mk II could easily resolve all of the fluorescent polystyrene nanospheres clearly on fluorescence and subsequently on 405 nm SSC and 785 nm SSC, this was not the case for the non-fluorescent silica nanospheres, all of which drowned in the excessive background on the two SSC parameters (Figure 2D). In order to reduce the amount of background, we attempted several approaches, such as discriminating background from true nanosphere events in different combinations of bivariate plots of a range of calculated features, including but not limited to the aspect ratio, mask area, gradient root mean square (RMS), unmasked channel intensity, Haralick texture features and different masking strategies (data not shown). However, we found that the best and most elegant approach to circumvent this issue was to identify silica nanospheres on a bivariate scatter plot of non-fluorescent events on 405 nm SSC vs 785 nm SSC (Figure 2E). By using this approach, it was possible to identify all of the silica nanospheres present in the mixture. However, they were located within a significant background of unknown origin, which was present only to a lesser degree in pure buffer samples. In addition, 100 nm silica nanospheres could be discriminated from the background based on their fluorescence (Figure 2H), which was in line with what was observed for the fluorescent polystyrene nanospheres. ImageStream X Mk II could not quantify the 100 nm and 180 nm silica beads with an adequate reproducibility (CV = 48.8% and 29.5%, respectively; Figure 3C). Although ImageStream X Mk II could quantify 240–590 nm silica nanospheres reliably (CV = 8.9–11.7%), greater variability was observed for 880 nm and 1300 nm silica nanospheres (CV = 24.3 and 28.8%, respectively). Interestingly, 110 nm and 500 nm polystyrene nanospheres could be quantified reliably (CV = 4.5 and 8.4%); however, greater variability was observed for the 280 nm population (CV = 25.4%).

Thus, using the optimal triggering threshold and gating strategies, Apogee A60 Micro-PLUS and ImageStream X Mk II had the lowest LoDs by being able to detect the smallest silica nanospheres, followed by FACS Aria III. In being fluorescent and thereby discriminable from the background, 110 nm fluorescent polystyrene nanospheres could be detected by both FACS Aria III, thereby resulting in a slightly lower functional LoD for this platform than determined with silica nanospheres. Apogee A60 Micro-PLUS had the lowest LoQ by being able to quantify all bead populations with adequate reproducibility. This was followed by ImageStream X Mk II, as determined with silica nanospheres. In line with observations for LoD, ImageStream X Mk II had a lower functional LoQ when assessed by polystyrene nanospheres due to their fluorescent properties, making them discriminable from the background. FACS Aria III could not quantify any of the nanospheres used in the mixture with adequate reproducibility.

#### 3.1.2. Quantifying Nanospheres in Practice

Although LoD and LoQ are useful to define the smallest particles that can be detected and discriminated from the background and quantified with satisfactory accuracy, respectively, they do not convey the ability of a cytometer to detect particles of different sizes. Therefore, we compared the concentrations of the different nanospheres between platforms in order to address this problem (Figure 2H).

When assessing the ability to detect small silica nanospheres (100–300 nm), Apogee A60 Micro-PLUS clearly outperformed both ImageStream X Mk II. Apogee A60 Micro-PLUS detected 24.6-fold more of the 100 nm population (*p* < 0.01), 1.4-fold more of the 180 nm population (*p* = 0.056, N.S.), 1.7-fold more of the 240 nm population (*p* < 0.05) and 1.7-fold more of the 300 nm population (*p* < 0.05) compared to ImageStream X Mk II. Although it could be argued that background could significantly contribute to the concentration of 100 nm silica nanospheres measured on Apogee A60 Micro-PLUS due to them not being able to be fully resolved, this was verified not to be the case by assessing the concentration of pure buffer and the nanosphere mixture lacking 100 nm silica nanospheres in the 100 nm population gate (Figure S7). Interestingly, FACS Aria III detected more of the 300 nm populations compared to all of the other platforms (Apogee A60 Micro-PLUS: 1.6-fold, *p* < 0.05; ImageStream X Mk II: 2.7-fold, *p* < 0.05). Although the underlying reason for this difference is somewhat unclear, a possible explanation could be that a proportion of the 240 nm nanospheres in the mixture was detected in the same region as 300 nm nanospheres on FACS Aria III due to a lack of sensitivity and ability to resolve these particles.

In contrast to the small silica nanospheres, differences between FACS Aria III, Apogee A60 Micro-PLUS and ImageStream X Mk II were less pronounced for the larger silica nanospheres (590 nm, 880 nm and 1300 nm) with the only statistically significant difference being in the concentration of the 590 nm population between Apogee A60 Micro-PLUS and ImageStream X Mk II (1.2-fold, *p* < 0.05).

Similar tendencies were observed for fluorescent polystyrene nanospheres. As with small silica nanospheres, Apogee A60 Micro-PLUS could detect more of the 110 nm population than FACS Aria III (1.5-fold, *p* < 0.05) and ImageStream X Mk II (1.8-fold, *p* < 0.05). All platforms detected similar concentrations of the 280 nm and 500 nm population.

In line with observations for LoD and LoQ, Apogee A60 Micro-PLUS tended to be more sensitive in detecting small silica nanospheres, scattering very small amounts of light than all other platforms, followed by ImageStream X Mk II and, lastly, FACS Aria III. For larger silica nanospheres, all platforms performed similarly. Finally, results for fluorescent polystyrene nanospheres reflected those for silica nanospheres, where Apogee A60 Micro-PLUS performed better than other platforms in detecting small nanospheres.

### 3.2. Detection of Different EV Phenotypes in Blood Plasma

**Figure 4:**
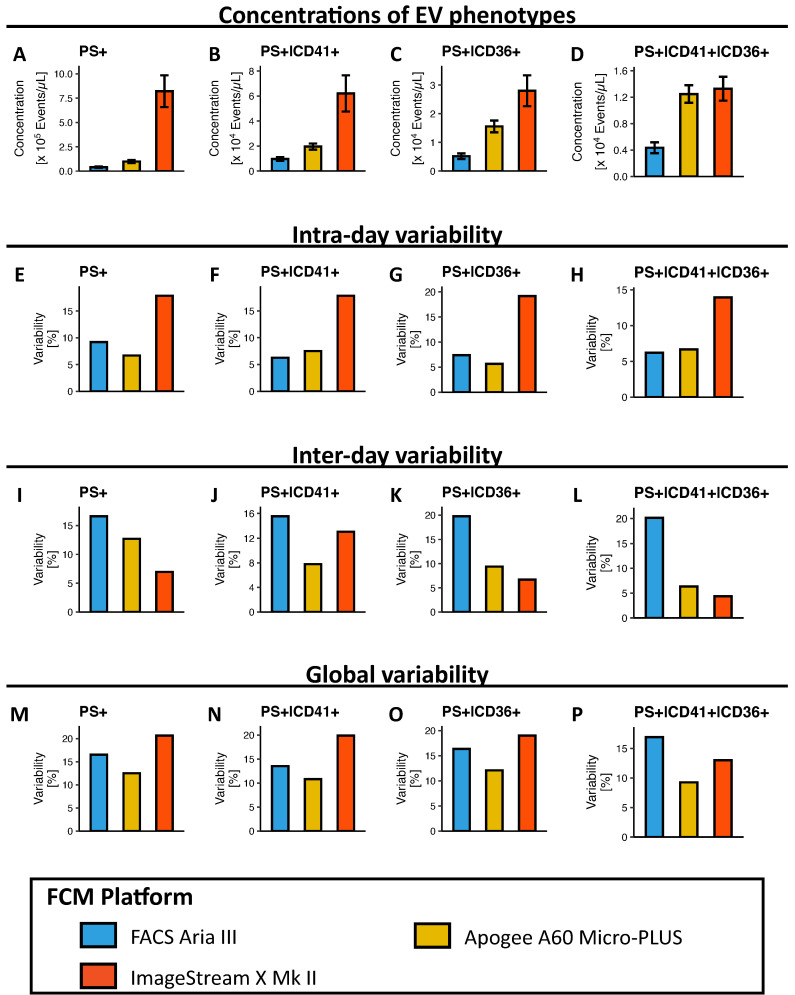
Concentrations and variability in the detection of EV phenotypes on three different flow cytometry platforms. (**A**–**D**) Concentrations of (**A**) PS+, (**B**) PS+|CD41+, (**C**) PS+|CD36+ and (**D**) PS+|CD41+|CD36+ EVs detected on the three different platforms. Results are depicted as mean (bar) ± 1SD (error bars) of concentrations from the inter-day variability study. (**E**–**H**) Intra-day variability in the detection of (**E**) PS+, (**F**) PS+|CD41+, (**G**) PS+|CD36+ and (**H**) PS+|CD41+|CD36+ EVs (*n* = 20 per platform). (**I**–**L**) Inter-day variability in the detection of (**I**) PS+, (**J**) PS+|CD41+, (**K**) PS+|CD36+ and (**L**) PS+|CD41+|CD36+ EVs (*n* = 5 × 5 per platform). (**M**–**P**) Global variability in the detection of (**M**) PS+, (**N**) PS+|CD41+, (**O**) PS+|CD36+ and (**P**) PS+|CD41+|CD36+ EVs (*n* = 40 per platform). Coefficients of variability (CV) were calculated as SD/mean expressed in percent.

One of the main aims of this study was to assess the ability of the different FCM platforms to detect and characterise different EV phenotypes directly in complex bio-fluids. To this end, 40 aliquots of the same PPP pool were stained directly with antibodies against EV markers expressed to varying degrees, defining four different phenotypes, and measured on each platform over the course of five analysis days (Figure 1E–H). The global mean concentrations of all samples for each phenotype were then statistically compared between platforms (Figure 4A–D).

Regarding the detection of the single-labelled, EV-defining PS+ EV phenotype (Figure 4A), ImageStream X Mk II detected a 21-fold greater concentration compared to FACS Aria III (*p* < 0.001) and 8.3-fold greater concentrations compared to Apogee A60 Micro-PLUS (*p* < 0.001). The Apogee A60 Micro-PLUS detected 2.6 (*p* = 0.008) more EVs than FACS Aria III.

Concerning the easily discriminable double-labelled PS+|CD41+ phenotype (Figure 4B), differences between ImageStream X Mk II and other platforms were less pronounced albeit somewhat comparable to the results on PS+ EVs, detecting 6.2-fold greater concentrations compared to FACS Aria III (*p* < 0.001) and 3.2-fold more than Apogee A60 Micro-PLUS (*p* < 0.001). Apogee A60 Micro-PLUS detected 2.0-fold more PS+|CD41+ EVs than FACS Aria III (*p* < 0.001). Somewhat similar results were obtained for the less discriminable PS+|CD36+ double-labelled phenotype (Figure 4C). ImageStream X Mk II detected 5.2-fold greater concentrations than FACS Aria III (*p* < 0.001) and 1.7-fold greater than Apogee A60 Micro-PLUS (*p* < 0.001). The Apogee A60 Micro-PLUS detected a 3-fold greater concentration than FACS Aria III (*p* < 0.001).

Comparing the ability of the different platforms to detect the triple-labelled PS+|CD41+|CD36+ EV phenotype (Figure 4D), ImageStream X Mk II only detected slightly more EVs than Apogee A60 Micro-PLUS (1.1-fold, *p* = 0.003), whereas ImageStream X Mk II could detect 2.9-fold more EVs than FACS Aria III (*p* < 0.001). Apogee A60 Micro-PLUS could detect 2.7-fold more EVs with this phenotype than FACS Aria III (*p* < 0.001).

Thus, taken together, ImageStream X Mk II could detect higher concentrations of EV phenotypes than all other platforms with the exception of triple-labelled EVs, for which Apogee A60 Micro-PLUS could detect similar concentrations. Finally, the Apogee A60 Micro-PLUS could detect higher concentrations of all EV phenotypes compared to FACS Aria III.

### 3.3. Variability in Determining EV Concentrations and Fluorescence Signals

Another main point of focus for this study was determining the variability in quantifying different EV phenotypes in bio-fluids on the four platforms. We assessed intra-day, inter-day and global variability, as all three parameters describe different aspects of stability in measurements (i.e., measurement error during a single analysis day vs from one day to another vs throughout a study).

Intra-day variability was investigated by labelling 20 individual aliquots of the same PPP pool and analysing the samples on a single analysis day, which would represent a medium to long analysis day (Figure 4E–H). ImageStream X Mk II presented with noticeably higher intra-day variabilities for all four EV phenotypes (CV = 13.9–19.2%) compared to all other platforms. Comparable variabilities were observed for FACS Aria III (CV = 6.2–9.2%) and Apogee A60 Micro-PLUS (CV = 5.7–7.5%).

Inter-day variability was evaluated by labelling and analysing five individual aliquots per day for five days on each platform and calculating the variability in daily means of samples (Figure 4I–L). Although ImageStream X Mk II had high intra-day variabilities for all phenotypes, it presented with the lowest overall inter-day variabilities (CV = 4.4–13.1%). Apogee A60 Micro-PLUS demonstrated similar or only slightly increased inter-day variabilities for all phenotypes compared to ImageStream X Mk II (CV = 6.3–12.7%). However, FACS Aria III (CV = 10.9–15.0%) presented with markedly increased overall inter-day variabilities compared to the other platforms.

In addition to intra- and inter-day variability, global variability was evaluated to simulate the systemic measurement error incurred over multiple analysis days, as would be the case for samples from most study cohorts. Global variability was assessed by calculating the coefficient of variability for all 40 aliquots individually labelled throughout the duration of this study (Figure 4M–P). Unsurprisingly, global variabilities tended to be higher for all platforms compared to intra- and inter-day variabilities. However, the magnitude varied between platforms. ImageStream X Mk II had the highest overall global variability of all platforms in measuring all phenotypes (CV = 13.0–20.7%), followed by FACS Aria III (CV = 13.5–16.9%). Finally, Apogee A60 Micro-PLUS had the lowest global variabilities in determining all of the four phenotypes (CV = 9.3–12.5%).

In summary, the Apogee A60 Micro-PLUS demonstrated the overall lowest variabilities in EV concentration determinations, and global and inter-day variabilities did not differ much from intra-day variabilities. The FACS Aria III performed similarly and exhibited somewhat increased variabilities in EV concentration determinations compared to the Apogee A60 Micro-PLUS. Finally, although the average concentrations of EV phenotypes did not differ between analysis days on the ImageStream X Mk II, it presented with the highest intra-day and global variabilities in EV concentrations across EV samples and phenotypes.

## 4. Discussion

Since the early days of the field of research into EVs, flow cytometry has been a popular method for their characterisation, as it allows for simultaneous detection of multiple parameters on single EVs. While conventional flow cytometry is still commonly used despite the bulk of EVs being below its lower detection limit, the increasing availability and affordability of high-resolution and imaging platforms more sensitive to the detection and characterisation of small particles have seen the use of these becoming more prevalent. Although all of these platforms have been available to researchers for some time, few direct benchmarks have been published, and the extent of published benchmarks is limited to the smallest size of synthetic nanospheres the platform can detect and discriminate.

In this study, the FACS Aria III has served as a comparator for the other platforms to verify their applicability in the detection of small particles and whether these platforms provide increased capabilities for characterising single EVs in complex biological fluids. The limitations of conventional flow cytometry represented by the FACS Aria III in this study in detecting and characterising single EVs has been discussed quite elegantly and thoroughly by other authors, and little more can be contributed to this discussion [20,21,22,24,41]. Therefore, the following sections have focussed on discussing the other platforms utilised in this study.

### 4.1. Detection of Synthetic Nanospheres

The use of synthetic nanospheres is somewhat contentious due to their physical properties often differing significantly from those of biological EVs. However, due to their availability, uniform nature and being inexpensive, both silica and polystyrene nanospheres are still being used for characterising the sensitivity and detection limits of FCM platforms and are additionally used as controls and for quality assurance (QA) [25,29,30,39,42,43]. These properties allow for the inclusion of several nanosphere populations with differing sizes, refractive indices, fluorescent properties or surface treatments to be included into control and QA regimens. Regardless, there has been some discussion about the applicability of synthetic nanospheres as reference controls for EVs due to their refractive indices being far above that of EVs [41,44]. At the commonly used illumination wavelengths used for light scatter measurements in modern flow cytometers, polystyrene nanospheres commonly used in characterising FCM sensitivity have a refractive index of 1.59–1.63 [45,46]. Furthermore, although silica nanospheres have a significantly lower refractive index of 1.45 [41,45,46,47], this is still somewhat higher than EVs, which have been estimated to have a refractive index below 1.40 [44]. Thus, depending on the particle size, illumination wavelength and the optical set-up of the FCM platform, polystyrene and silica nanospheres would scatter between 40 and 300 or 5 and 50-fold more light than EVs, respectively [46,47,48]. While several synthetic and biological alternatives (i.e., liposomes, virus particles and hollow core-in-shell nanospheres) have been suggested as alternatives, these either come with their own issues, such as non-uniformity, low reproducibility and traceability, biological hazards or unavailability to the general research community. Thus, we opted to use a combination of silica and polystyrene nanospheres in this study to characterise platform sensitivity, as their uniform nature allows for easy detection and discrimination on either single parameter histograms or multi-parameter scatterplots. In order to make the comparison as thorough and relevant as possible, we decided to define the lower LoD of each platform based on the smallest detectible nanosphere population. Unlike previous studies that defined the lower LoD on the smallest nanosphere discriminable from other nanospheres and background on a single scatter parameter [24,25,29,39], we defined the lower LoD based on the best combination of parameters for each platform. While it could be argued that this is not completely comparable due to potentially differing data analysis strategies, experienced cytometrists would utilise data analysis strategies that suit their respective platforms best and thereby gain additional sensitivity that is unattainable in a direct comparison. In that sense, we argue that this comparison could be considered closer to reality.

In this study, only the Apogee A60 Micro-PLUS and the ImageStream X Mk II were able to detect all of the nanospheres used to determine LoD. Although much of this sensitivity on both methods stem from slow sheath speeds, tight sample cores and high magnification of the interrogation area, there are some distinct differences between these platforms.

Much of the sensitivity of the Apogee A60 Micro-PLUS is derived from a combination of the slow sheath and sample flow rates, usage of powerful lasers for illumination of particles in the sample core, confocal high-magnification of the interrogation zone and several inline particle filters to remove impurities in the sheath fluid. Together, these strategies have been shown to increase the intensity of signals captured from particles in the sample, while decreasing the amount of background signals from impurities in the sheath fluid and Mie and Raleigh light scatter of laser light by particles or molecules outside the focal plane [20,21,22]. According to Mie and Raleigh’s theories on light scattering by small particles, light scatter is somewhat dependent on the wavelength of the incident light. A significant factor to consider in this regard is the functional limit where the size of a particle determines whether a particle transitions predominantly, conforming to one of these theories to the other. While this limit has traditionally been set to particles much smaller than the wavelength of the incident light, theoretical and physical experiments have demonstrated that this limit is at particles with a diameter around one-tenth of the wavelength of incident light [40]. Thus, the use of a 405 nm laser for light scatter instead of the 488 nm lasers typically used in flow cytometers could contribute to the theoretical lower limit of detection being reduced. Whether this has an effect on the detection and discrimination of particles from the background is much more complex, however, and other factors might contribute more to the functional sensitivity of a cytometer. First, it is widely accepted that higher laser power also results in larger signals and separation between background and true particles, which has been demonstrated for both fluorescence [49] and light scatter measurements from small particles [Botha et al., in preparation]. Thus, by utilising lasers with significantly higher power ratings than conventional systems (180–300 mW versus 3–50 mW), the sensitivity of the Apogee A60 Micro-PLUS is further increased. Second, a factor that is often overlooked by cytometrists in improving and discussing sensitivity to detect and discriminate particles on light scatter on a flow cytometer is the collection angles of scattered light. Although this is a complex subject, it has been demonstrated that collecting light in an angle between conventional FSC and SSC could improve both the sensitivity of detecting light scatter from particles and to discriminate them from the background [20,21]. As such, by not only acquiring signals in the MALS channel on the Apogee A60 Micro-PLUS but also setting the triggering threshold in this channel, it was possible to reduce the amount of background acquired in general while acquiring dim signals from small particles.

Unlike conventional flow cytometers that utilise PMTs, the ImageStream X Mk II utilises highly sensitive CCDs for signal detection. Although PMTs have a much higher dynamic range, CCDs exhibit much higher quantum efficiencies (typically 40–95% depending on their design and the wavelength of light detected versus 20% for most PMTs) and also a markedly lower rate of dark counts (<0.001 versus 4 electrons/pixel/second) [50]. By using TDI technology, in which pixel intensities for each particle in each channel are integrated for all time points the particle is present in the field of view of the CCD, the limited dynamic range of CCDs can be improved. With this technology, true signals can be amplified far above that of background, as true particles would have a much more stable emission or scatter pattern than the background. In addition to this, triggering on the ImageStream X Mk II occurs in all channels on all signals above CCD background, thereby limiting rejection of potential true signals. Together, these features have previously been demonstrated to enable detection of 20–40 nm fluorescent polystyrene nanospheres on the ImageStream X Mk II [25]. Although it has been suggested based on Mie light scattering theory that the usage of a 405 nm laser would improve the sensitivity of the ImageStream X Mk II in detecting small particles, this was not the case in this study. Light scatter signals collected from the 405 nm laser did not allow for better separation of populations from each other nor from the background, and the number of background events was generally higher when collecting light scatter signals from the 405 nm laser. While this result could seem somewhat contrary to theory, identification of true signals is often a complex interplay between the relative levels of signal to noise. Therefore, removing an optical filter would hardly be enough to distinguish between signals and noise, and additional optimisation, including focussing of the laser and confocal light collection, would be necessary. Besides, laser alignment is very important in this case, as a slightly misaligned laser might cause diminished signals from true particles, while additional noise could be introduced. However, although somewhat problematic, SSC from the 405 nm laser did allow for successful discrimination of the non-fluorescent silica nanospheres from the background when used in combination with SSC from the 785 nm laser, thereby attributing to the low LoD of the ImageStream X Mk II.

### 4.2. Quantification of Synthetic Nanospheres

Apart from LoD, we additionally compared the reliability of platforms in detecting nanospheres of different sizes and termed the smallest nanosphere population quantified with a CV of less than 20% as the functional LoQ. In addition to this, the variability in concentration determination for each bead population was measured, which could give an indication as to the reproducibility of measurements of particles of different sizes. This metric additionally highlights inconsistencies in detecting particles with different physical characteristics on each individual platform, which could also provide valuable information as to the sensitivity, potentials and limitations of platforms in detecting and characterising EVs, as being able to detect a certain nanosphere population and doing so reproducibly are two different things entirely.

In this study, the Apogee A60 Micro-PLUS had the lowest LoQ of all platforms tested, yielding concentration variabilities between 3.9% and 9.9% for all detected nanospheres. Much of this high reproducibility could be attributed to the same factors that contribute to the sensitivity of this platform, as described above. Additionally, the usage of a syringe pump equipped with a highly standardised volumetric syringe (1700 series Hamilton^®^ GASTIGHT^®^ syringe, Hamilton Company, Reno, NV, USA) for driving the sample combined with slow sample flow rates (0.75 µL/min) also contributed to the accuracy and reproducibility of concentration determinations. In addition to having the lowest LoQ of all the platforms tested in this study, the Apogee A60 Micro-PLUS was also able to detect significantly larger concentrations of the smallest silica and polystyrene nanospheres, which could be attributed to all of the above-mentioned features that contribute to the sensitivity and reproducibility of this platform. Of note, both the use of the MALS channel for detection and triggering and approaches to reduce noise are main factors, enabling the Apogee A60 Micro-PLUS to detect small nanospheres efficiently and reproducibly.

Even though the ImageStream X Mk II had a comparable LoD to the Apogee A60 Micro-PLUS, its LoQ was considerably higher, and significant variability was observed for very small and very large non-fluorescent silica nanospheres. As mentioned previously, much of this variability could be attributed to the amount of non-specific background events in the same region as the nanospheres, which, in turn, could be attributed to the use of the 405 nm laser for light scatter determinations by removing the notch filter. Thus, although little background was observed in pure buffer samples, the combination of using the 405 nm laser for scattering in this manner and the beads in buffer with added detergent could result in the acquisition of variable amounts of background events. When looking at the fluorescent polystyrene nanospheres, however, variabilities were much lower as these beads were completely discriminable from the background, further strengthening this hypothesis. When comparing the efficiency at which nanospheres with different sizes could be quantified, the ImageStream X Mk II had a tendency to detect fewer of the smaller nanospheres than the Apogee A60 Micro-PLUS, regardless of composition or fluorescence properties. Although this result was somewhat surprising, there could be a logical explanation for this in the physical properties and limitations of the optical system. In the present study, the sample core diameter was set to the minimum of 6 µm to keep it stable and the largest amount of particles in focus. This, however, was much larger than what the optics allow to be in focus, which would mean that large proportions of particles would be out of focus. This would particularly affect smaller particles with dim light scatter and fluorescence signals, as their signals would become more dispersed the further they are away from the focal point. This could explain why the ImageStream X Mk II detected fewer of the smaller nanospheres than the Apogee A60 Micro-PLUS, while the two platforms tended to detect equivalent concentrations of the larger particles. While this would not have much of an impact on particles with large scatter profiles or with high fluorescence values, small particles with either no or low fluorescence signals would have significantly lower measurable signals due to becoming more diffuse and, in some cases, reaching levels equivalent to background light. This was somewhat verified in the results of this study, where concentrations of particles increased with either increasing size for non-fluorescent nanospheres or by adding fluorescence in the case of 110 nm polystyrene nanospheres that scatter slightly less light than 180 nm silica nanospheres (Figure 2G). In order to circumvent this issue, the ImageStream X Mk II has a combined hardware and software feature that provides an extended depth of field (EDF). While EDF could theoretically extend the DOF significantly and is often used when analysing cells, others have shown that this feature does not provide an advantage in detecting small particles, as fewer particles tend to be detected with EDF-enabled [29]. Another explanation for the ImageStream X Mk II detecting lower concentrations of the smaller nanospheres could be because the object detection thresholds and masking algorithms on the ImageStream X Mk II were designed for cells and cell-sized objects. Based on this, very small dim objects might not reach the requirements to be an object according to the INSPIRE software, thereby excluding them from the data.

### 4.3. Characterisation of EVs in Blood Plasma

While measuring synthetic nanospheres provide valuable information on the raw performance of FCM platforms in characterising particles within the EV size range albeit, with some reservations regarding differing physical properties, EV preparations are usually much more complex owing to their biological origin. In order to reduce the complexity of samples containing biological EVs, researchers have employed a whole range of different purification techniques, including differential centrifugation, density gradient centrifugation, ultra-filtration, size exclusion chromatography, polymer-based purification, affinity purification methods. However, in order to limit the amount of pre-analytical variability introduced in sample handling and storage, we chose to characterise EVs directly in blood plasma in this study, which is a preferred practice for some research groups in characterising single EVs using flow cytometers aimed at keeping the method as clinically applicable as possible [47,51,52,53]. We compared the ability of the different platforms to detect four different EV phenotypes with focus on concentrations of each respective phenotype and its intra-day, inter-day and global variability.

Even though the Apogee A60 Micro-PLUS had the lowest LoQ of all platforms included in this study and a comparable LoD to the ImageStream X Mk II, it detected significantly fewer single and double-labelled EV phenotypes than the ImageStream X Mk II. The most likely explanation for this result is that excessive background stemming from the samples causes some of the truly positive EVs to be excluded from the analysis. While the optical configuration of the Apogee A60 Micro-PLUS has been highly optimised to increase signals from samples while reducing much of the methodological background, it is almost impossible to account for other entities in the sample using conventional PMT signal detection. This would be especially evident in complex samples like blood plasma, where several different entities share similar physicochemical properties with EVs, including lipoproteins and protein aggregates. These observations have also been made by other researchers previously [7], and it has been demonstrated that a large proportion of events detected on an Apogee flow cytometer are, in fact, lipoproteins [47]. Remarkably, despite the high background, little variability was observed in concentrations of the different EV phenotypes investigated in this study. This is likely because the control samples and the actual samples were affected in similar ways, which meant that background could be successfully removed from the analysis, thereby yielding stable concentrations of EVs.

In the present study, the ImageStream X Mk II detected far greater concentrations of single-labelled PS+, double-labelled PS+|CD41+ and PS+|CD36+ EVs than all other platforms, while it also detected significantly more of the triple-labelled PS+|CD41+|CD36+ EVs than the FACS Aria III. One of the main advantages of the ImageStream X Mk II in detecting and characterising small particles was its ability to trigger on all channels simultaneously. By doing so, the ImageStream X Mk II was able to acquire data on all particles that had a signal above the camera level in at least one channel, which reduced the risk of true particles being excluded from data analysis. An additional advantage in the detection of EVs was using light scatter from the 405 nm laser, as this increased the probability of particles having a signal in this channel above camera background. Thus, in cases where FITC from phosphatidylserine expression alone was not enough to trigger as an object, one of the other fluorescent markers or the 405 nm scatter likely had enough signal for the particle to be triggered and collected. Therefore, even dim particles could be included in the analysis, which likely gave the ImageStream X Mk II its superior sensitivity. Even though the ImageStream X Mk II had the lowest inter-day variability of all platforms, its intra-day and global variabilities in concentration determinations of EVs were some of the highest. Although the precise nature of this is unknown, several possible explanations could be considered. First, although collecting light scatter from the 405 nm laser improved sensitivity, it also introduced a considerable background, which could affect samples in a variable manner. Second, the speed beads being utilised for synchronising the two CCDs consisted of polystyrene, which had a much higher refractive index than most EVs, which also affected its ability to scatter light. By combining high laser powers with highly sensitive CCDs, light scattered from these particles could have overshadowed some EVs, which were later excluded from the analysis by being gated out as artefacts. Third, during each data acquisition, the ImageStream X Mk II synchronised and focussed the sample core between the two CCDs. While this would not be too problematic under normal circumstances when measuring cells, the sample core width and flow speed were decreased significantly in this study, which meant that some stability was sacrificed on behalf of sensitivity. Thus, by having a slightly unstable sample core and signal integration times between samples, additional variability could be conferred to concentration determinations. Finally, although all of the sample preparations were performed by the same investigator, it could not be excluded that some portion of this variability could stem from pre-analytical procedures, which were only noticeable by virtue of the sensitivity of the ImageStream X Mk II.

Comparing the EV concentrations measured on the three different platforms to the true physiological concentration of EVs is no simple task. Although EVs (exosomes and microvesicles) in various biofluids have been measured quite extensively throughout the past decade, there is little agreement in the literature concerning the concentration of EVs in blood plasma from healthy individuals with concentrations ranging from as little as 200 to as much as 10^9^ EVs/µL [1,54]. Much of this disagreement stems from lack of sensitivity on certain platforms (cFCM) to detect small particles, yielding relatively low concentrations, while others are more sensitive to contaminating factors, such as various species of lipoproteins and protein aggregates (nanoparticle tracking analysis, atomic force microscopy), thereby yielding very high concentrations and skewed particle size distributions. In this study, the FACSAria III was able to detect a median 3.7 × 10^4^ PS+ EVs/µL, whereas the Apogee A60 Micro-PLUS was able to detect 1.01 × 10^5^ EVs/µL and the ImageStream X Mk II 8.59 × 10^5^ EVs/µL. Two independent studies by van der Pol et al. [14] and Arraud et al. [11] previously demonstrated that conventional flow cytometers were able to detect less than 1% of the bulk of EVs with a lower size cut-off of 270–300 nm as compared to quantitative immuno-TEM, which is considered by many to be the gold-standard in characterising EVs due to its ability to detect even the smallest particles in a sample (~1 nm) and despite the low sample throughput of this method [14] and operator dependency of results [55]. In addition, van der Pol et al. also demonstrated that a high-resolution flow cytometer only under-estimated the concentration of EVs in a biological sample by 15-fold with a significantly improved lower size cut-off of 150–190 nm compared to TEM, thus giving a 20-fold difference between conventional and high-resolution flow cytometry [14]. This was, however, not quite the case in this study, as the FACSAria III was able to detect approximately 37% of the PS+ EVs detected by the Apogee A60 micro-PLUS. One of the most apparent reasons for this could be that the ability of conventional flow cytometers to detect fluorescently labelled EVs could further be improved by up to 40-fold by using a fluorescence trigger rather than a scatter trigger [15], a strategy which the authors of the present study opted for. This strategy has also been estimated to improve the lower size cut-off of conventional flow cytometers to around 200 nm [16], which includes a significantly larger proportion of the bulk of EVs compared to the standard triggering strategy, as the concentration of EVs increases exponentially with decreasing size until a peak size of between 50 and 100 nm, after which it drops off with decreasing size [11,14]. It was, however, impressive that the ImageStream X Mk II detected 8.5-fold more PS+ EVs than the Apogee A60 Micro PLUS and 23.2-fold more than the FACSAria III. Much of this is due to the advantages of imaging flow cytometers described above, including using low noise CCDs, TDI of each particle, and detecting even dimly fluorescent particles due to triggering in all channels. How this translates to a lower size cut-off is uncertain, as no groups have yet made a head-to-head comparison between concentrations and detectable EV-size distributions obtained on imaging flow cytometers and other methods, such as NTA and TEM, to the authors’ knowledge. Previously, however, Headland et al. showed that the ImageStream X Mk II could detect fluorescent polystyrene beads as small as 20 nm, with almost no detectable light scatter signal based solely on the fluorescence of these nanospheres [25]. Although polystyrene beads have a much higher refractive index than EVs, this would probably not matter in this case, as light scattering off of a 20 nm polystyrene nanosphere would likely be too dim to detect even with a high-powered 405 nm laser. As such, the ImageStream X Mk II could theoretically detect the entire size range of EVs, provided that each EV is sufficiently fluorescent.

While describing the ability of a method to discover and detect EVs within a specific size and fluorescence range is certainly a useful metric, other properties related to the flow cytometric platform used are also important to consider when interpreting results. In this study, we demonstrated that it is necessary to describe the ability of a method to detect particles throughout the size and fluorescence range where EVs are expected to fall, as asymmetric recovery throughout this range or excessive variability within a certain region in this range might complicate data interpretation. In addition, it is also important to consider which methodological platform should be used for a specific study design, as the inherent properties of a certain platform might make it suitable for one application and unsuitable for another. Specifically, the Apogee A60 Micro-PLUS could determine EV concentrations very reproducibly and had the lowest intra-day and global variabilities of all tested platforms and acceptable intra-day variabilities as well. However, it detected far fewer EVs than the ImageStream X Mk II, which could partly stem from a significantly lower signal-to-noise ratio and being more sensitive to other factors in the sample, such as lipoproteins and protein aggregates. As such, the Apogee A60 Micro-PLUS would be more suitable for use in studies, where EV concentrations are compared between different study groups (i.e., clinical studies), while it would be less suitable in studies used for discovery of EV phenotypes or validation of whether a specific EV phenotype is present in a sample. On the other hand, the ImageStream X Mk II could detect far larger concentrations of EVs of all of the investigated phenotypes than all other platforms and could do so reproducibly with low inter-day variability in mean concentrations of EVs. However, it had large intra-day and global variabilities and, therefore, large dispersion in the concentrations of individual samples. Thus, the ImageStream X Mk II would be very suitable for discovering or validating whether a specific EV phenotype is present in a sample, while it might be less suitable for comparing EV phenotypes between different study groups due to the large variability in determining EV concentrations.

## 5. Conclusions

By applying this methodology presented in this study, different strengths and weaknesses of platforms can be elucidated. By not only defining the smallest particle a platform can detect but also the reproducibility of concentration determinations of several different particles with different physical properties (e.g., size, RI, fluorescence), the performance of a platform can be characterised across the entire range of parameters, where it is expected for EVs to be detected. By comparatively analysing the concentrations of these beads across several platforms, platforms’ specific issues can be discovered and perhaps be improved upon. Determining both relative EV concentrations and the variability in EV concentration measurements could also help in choosing the correct platform for a study. On the one hand, discovery-based or rudimentary characterisation studies might prefer the use of highly sensitive methods to detect large amounts of EVs for discovering or validating the presence of specific EV phenotypes in a sample. On the other hand, in clinical studies, where differences in EV concentrations between groups are either not known or expected to be small, platforms with higher reproducibility would be preferable, as these would have a larger potential at finding true differences between patient groups by not inferring overt methodological variability to sample concentration determinations.

In conclusion, this study contributes to a growing wealth of research that demonstrates the necessity for researchers to know their methods and the peculiarities of the platforms they use for EV characterisation. Although great advances have been made in standardising EV characterisation on flow cytometers during the past few years, knowing the limitations and possible solutions to these for each individual platform would aid tremendously in providing reproducible and reliable results. This study attempted to lay the ground for more robust and comprehensive comparisons of flow cytometry platforms for the characterisation of EVs. Although extensive, this methodology for comparing flow cytometers is far from perfect and can be improved upon in several areas, as great advances have been made by other researchers and designated standardisation committees in generating and reporting highly standardised results since the practical work in this study was concluded, and incorporating these into comparisons would only strengthen conclusions drawn from these results. Nonetheless, using this or similar methodology for characterising and comparing platforms would allow researchers to discover different limitations on their chosen platforms and aid them in matching suitable flow cytometry platforms with projects based on the needs of each individual project. This would contribute to improving on the reproducibility of studies and thereby increase the quality and robustness of results in EV studies, all of which would advance the field of EV research.

## Figures and Tables

**Table 1:** Antibody master mix for flow cytometric characterisation of EVs in a single 50 µL PPP sample.

Specific Antibody Sample Mix	Isotype Control Antibody Sample Mix
10 µL Lactadherin-FITC (83 µg/mL, DF 1×; Haematologic Technologies Inc., Essex Junction, VT, USA; Cat. no. HALOBLAC-FITC)	10 µL Lactadherin-FITC (83 µg/mL, DF 1×; Haematologic Technologies Inc., Essex Junction, VT, USA; Cat. no. HALOBLAC-FITC)
10 µL anti-CD41-APC (25 µg/mL, DF 1×; Clone HIP8; Biolegend, San Diego, CA, USA; Cat. no. 303710)	10 µL IgG1, κ-APC (200 µg/mL, DF 8×; Clone MOPC-21; Biolegend, San Diego, CA, USA; Cat. no. 400120)
5 µL anti-CD36-PE (200 µg/mL, DF 1×; Clone 5–271; Biolegend, San Diego, CA, USA; Cat. no. 336206)	5 µL IgG2a, κ-PE (200 µg/mL, DF 1×; Clone MOPC-173; Biolegend, San Diego, CA, USA; Cat. no. 400212)

Abbreviations: EVs: extracellular vesicles; PPP: platelet-poor plasma; FITC: fluorescein isothiocyanate; APC: allophycocyanin; PE: phycoerythrin; DF: dilution factor prior to the preparation of master mix.

## Data Availability

The data presented in this study are available in supplementary files 2–5. Raw flow cytometry data presented in this study are available on request from the corresponding author. Raw flow cytometry data are not publicly available due to the extensive file sizes of the data included in this study.

## Supplementary Materials

The following are available online at https://www.mdpi.com/2227-9059/9/2/124/s1, SupplementaryResults.docx: Supplementary Figures S1–S7; 20190411_AllFCM_Beads.xlsx: Extracted data for beads samples from all flow cytometers; 20180403_FACSAria.xls: Extracted data for PPP on FACSAria III; 20180415_Apogee.xls: Extracted data for PPP on A60 Micro-PLUS; 20190605_ISXII_Samples.txt: Extracted data for PPP on ImageStream X Mark II; 20201221_ComparisonStudy_BeadQuant.R: R-script for statistical data analysis and plotting of bead data; 20201221_ComparisonStudy_EVPhenotypeAnalysis.R: R-script for statistical data analysis and plotting of PPP data.
