# Supplementary material for: Conventional, High-Resolution and Imaging Flow Cytometry: Benchmarking Performance in Characterisation of Extracellular Vesicles
Source: Biomedicines. 2021 Jan 27;9(2):124. doi: 10.3390/biomedicines9020124 (PMC7911094; doi:10.3390/biomedicines9020124)
Supplement: Supplementary file 1 [file biomedicines-09-00124-s001.zip › SupplementaryResults.docx]

Conventional, high-resolution and imaging flow cytometry: Benchmarking performance in characterisation of extracellular vesicles

Supplementary Materials

Jaco Botha ^a,b^, Haley R. Pugsley ^c^, Aase Handberg ^a,b^

^a^ Department of Clinical Biochemistry, Aalborg University Hospital, North Denmark Region, Aalborg, Denmark

^b^ Department of Clinical Medicine, Aalborg University, Aalborg, Denmark

^c^ Luminex Corporation, Seattle, Washington, United States of America

**Corresponding author:**

Jaco Botha

[j.botha@rn.dk](mailto:j.botha@rn.dk)

Sdr. Skovvej 15, Room 309

DK-9000 Aalborg

North Denmark Region

Denmark


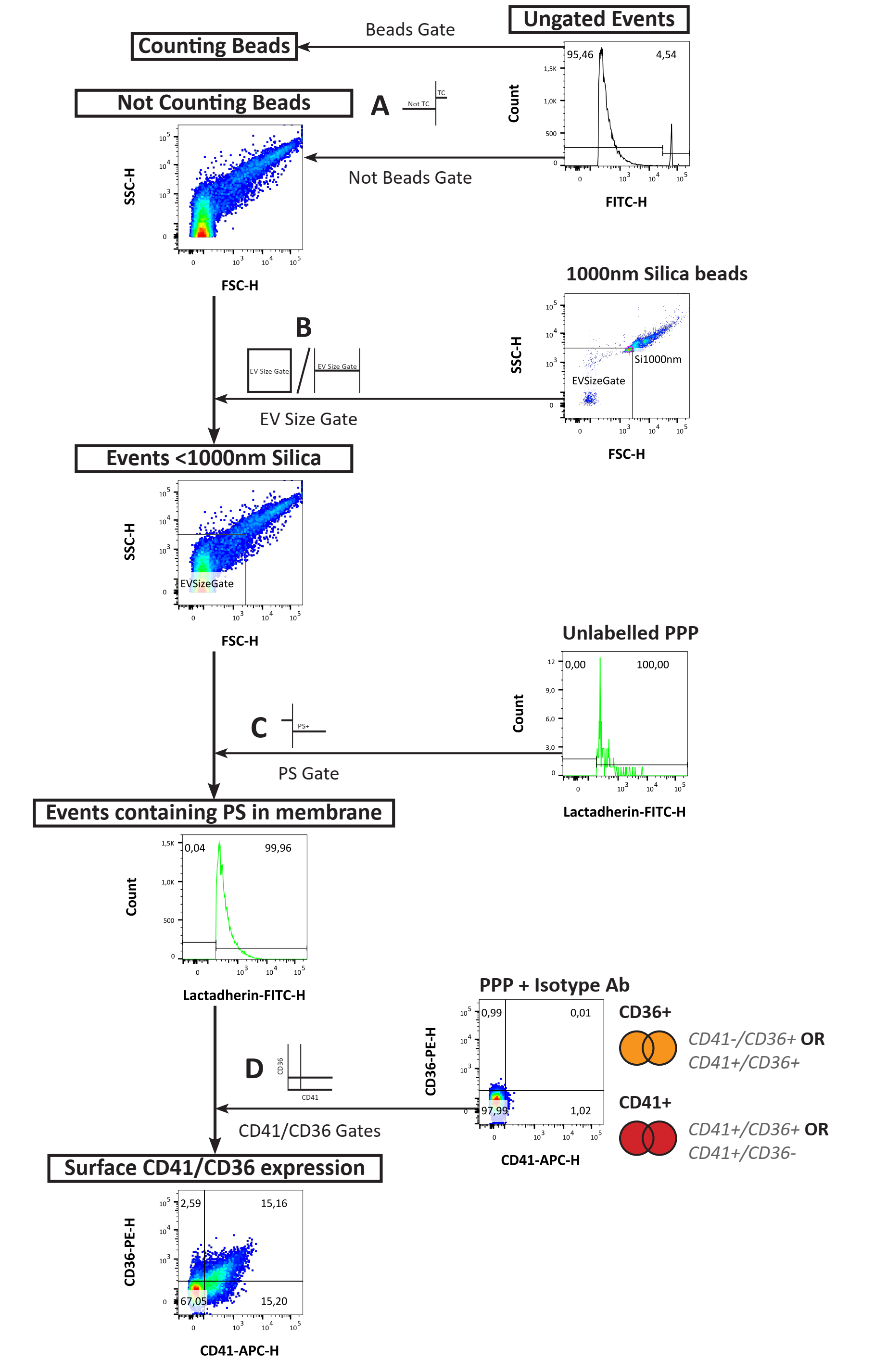


**Fig. S1: Specific EV-defining gating strategy for FACS Aria III. A)** First, a bi-sector gate was established to separate counting beads from other events in each sample. **B)** An EV size gate was established on the 99th percentile of green fluorescent 1000nm silica nanospheres (RI 1.4696 @ *λ* = 405nm) outside of the counting bead gate on FSC vs. SSC. **C)** On events within the EV size gate in an unlabelled PPP sample, a gate was set on the triggering threshold value of 200 to define lactadherin-binding phosphatidylserine (PS)+ events. **D)** On PS+ events in a PPP sample stained with isotype control antibodies, quadrant gates were established on the 99th percentiles of events in the APC vs. PE channels to define different combinations of CD41+/- and CD36+/- events, respectively. Finally, the sums of CD41+ and CD36+ events were defined by establishing logical OR gates on “*CD41+|CD36+ OR CD41+|CD36-*“ and “*CD41-|CD36+ OR CD41+|CD36+*”, respectively. Gates **A-D)** were transferred to all samples acquired on the flow cytometer during the same analysis day and done for each analysis day separately.


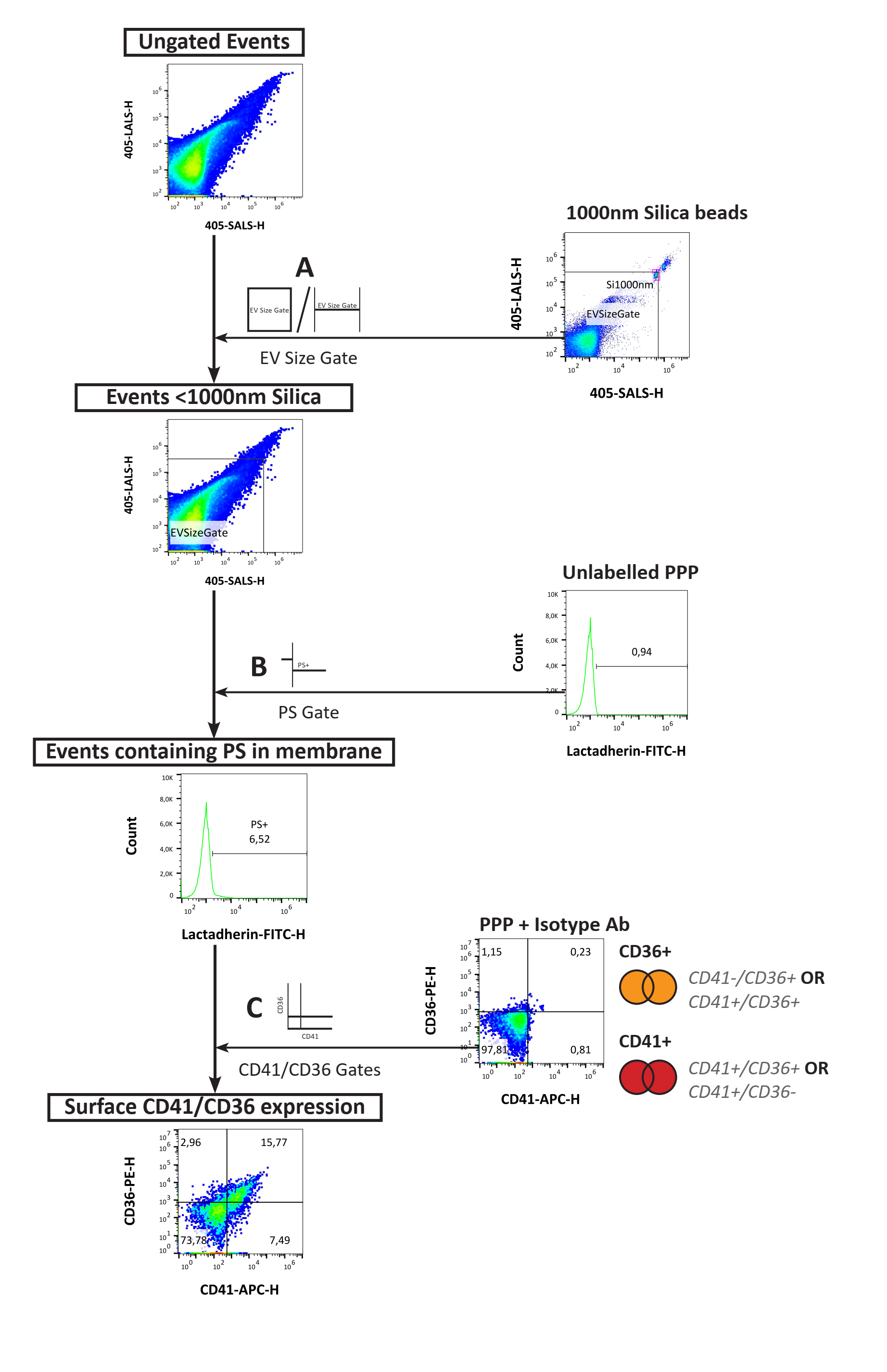


**Fig. S2: Specific EV-defining gating strategy for Apogee A60 Micro-PLUS. A)** An EV size gate was established on the 99th percentile of green fluorescent 1000nm silica nanospheres (RI 1.4696 @ *λ* = 405nm) outside of the counting bead gate on SALS vs. LALS. **B)** On events within the EV size gate in an unlabelled PPP sample, a gate was established on the 99th percentile in the FITC channel to define lactadherin-binding phosphatidylserine (PS)+ events. **C)** On PS+ events in a PPP sample stained with isotype control antibodies, quadrant gates were established on the 99th percentiles of events in the APC vs. PE channels to define different combinations of CD41+/- and CD36+/- events, respectively. Finally, the sums of CD41+ and CD36+ events were defined by establishing logical OR gates on “*CD41+|CD36+ OR CD41+|CD36-*“ and “*CD41-|CD36+ OR CD41+|CD36+*”, respectively. Gates **A-C)** were transferred to all samples acquired on the flow cytometer during the same analysis day and done for each analysis day separately.


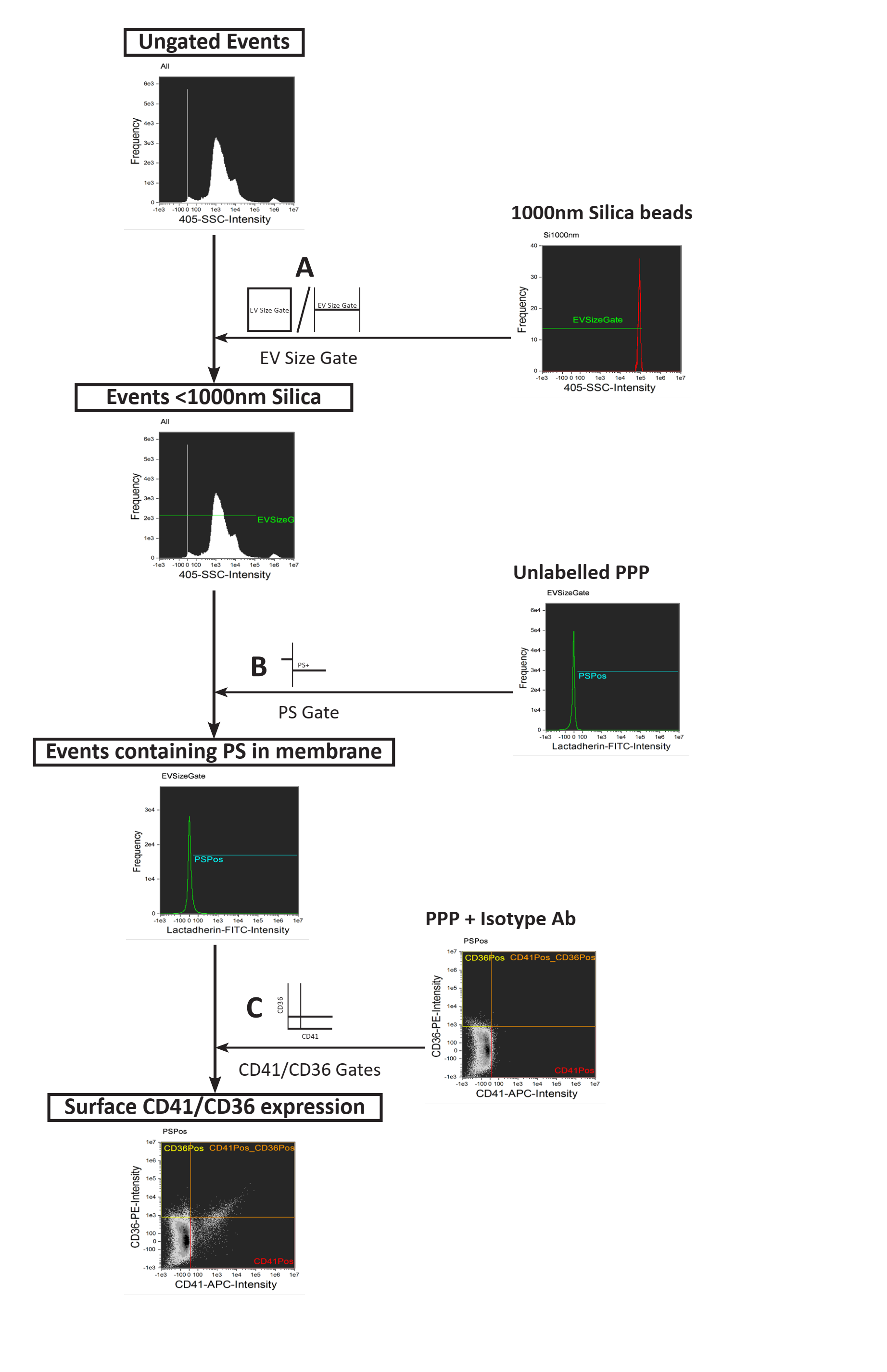


**Fig. S3: Specific EV-defining gating strategy for ImageStream X Mk II. A)** An EV size gate was established on the 99th percentile of green fluorescent 1000nm silica nanospheres (RI 1.4696 @ *λ* = 405nm) outside of the counting bead gate on 405nm SSC. **B)** On events within the EV size gate in an unlabelled PPP sample, a gate was established on the 99th percentile in the FITC channel above the green triggering threshold to define lactadherin-binding phosphatidylserine (PS)+ events. **C)** On PS+ events in a PPP sample stained with isotype control antibodies, quadrant gates were established on the 99th percentiles of events in the APC vs. PE channels to define different combinations of CD41+/- and CD36+/- events, respectively. Gates **A-C)** were transferred to all samples.


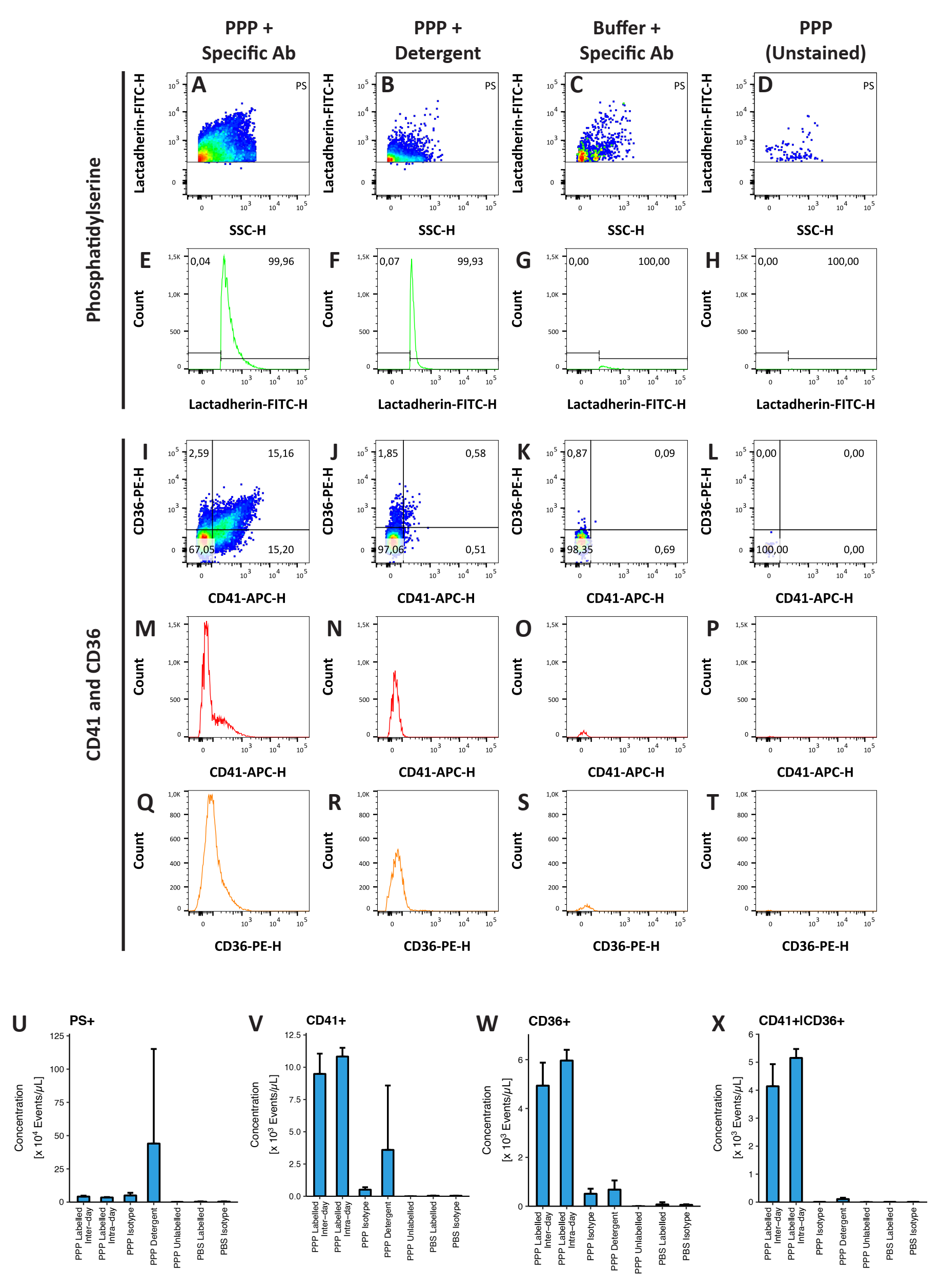


**Fig. S4: Controls for presence of EVs for data collected on FACS Aria III. A-T)** Scatterplots and histograms depicting differences in signals in the **A-H)** phosphatidyserine-FITC and **I-T)** CD41-APC and CD36-PE channels between **A, E, I, M, Q)** PPP stained with specific antibody mixture, **B, F, J, N, R)** stained PPP after lysing with Triton X-100, **C, G, K, O, S)** buffer labelled with specific antibody, and **D, H, L, P, T)** unstained PPP. **U-X)** Depiction of concentrations differences of **U)** PS+, **V)** PS+|CD41+, **W)** PS+|CD36+, and **X)** PS+|CD41+|CD36+ events.


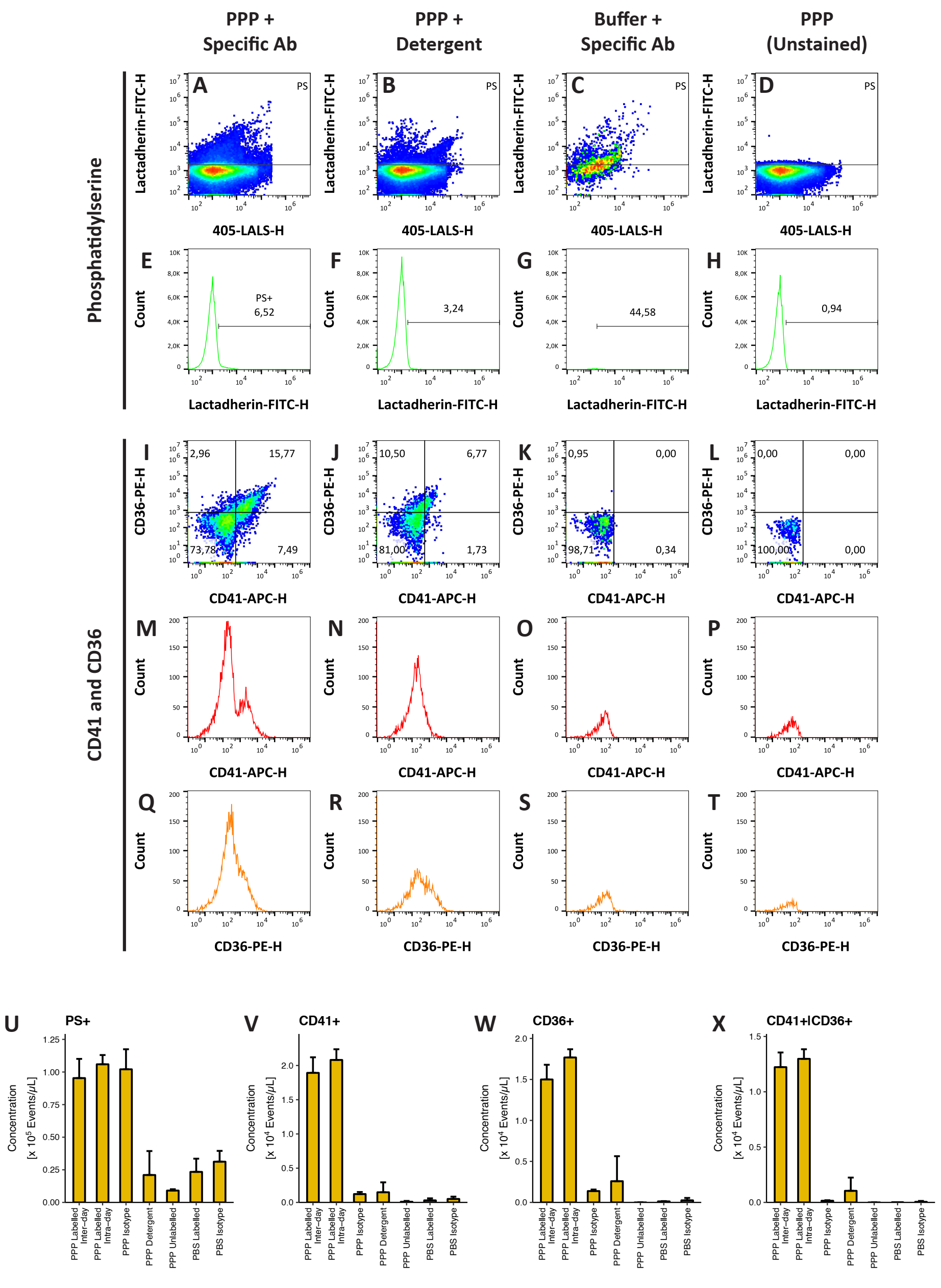


**Fig. S5: Controls for presence of EVs for data collected on Apogee A60 Micro-PLUS. A-T)** Scatterplots and histograms depicting differences in signals in the **A-H)** phosphatidyserine-FITC and **I-T)** CD41-APC and CD36-PE channels between **A, E, I, M, Q)** PPP stained with specific antibody mixture, **B, F, J, N, R)** stained PPP after lysing with Triton X-100, **C, G, K, O, S)** buffer labelled with specific antibody, and **D, H, L, P, T)** unstained PPP. **U-X)** Depiction of concentrations differences of **U)** PS+, **V)** PS+|CD41+, **W)** PS+|CD36+, and **X)** PS+|CD41+|CD36+ events.


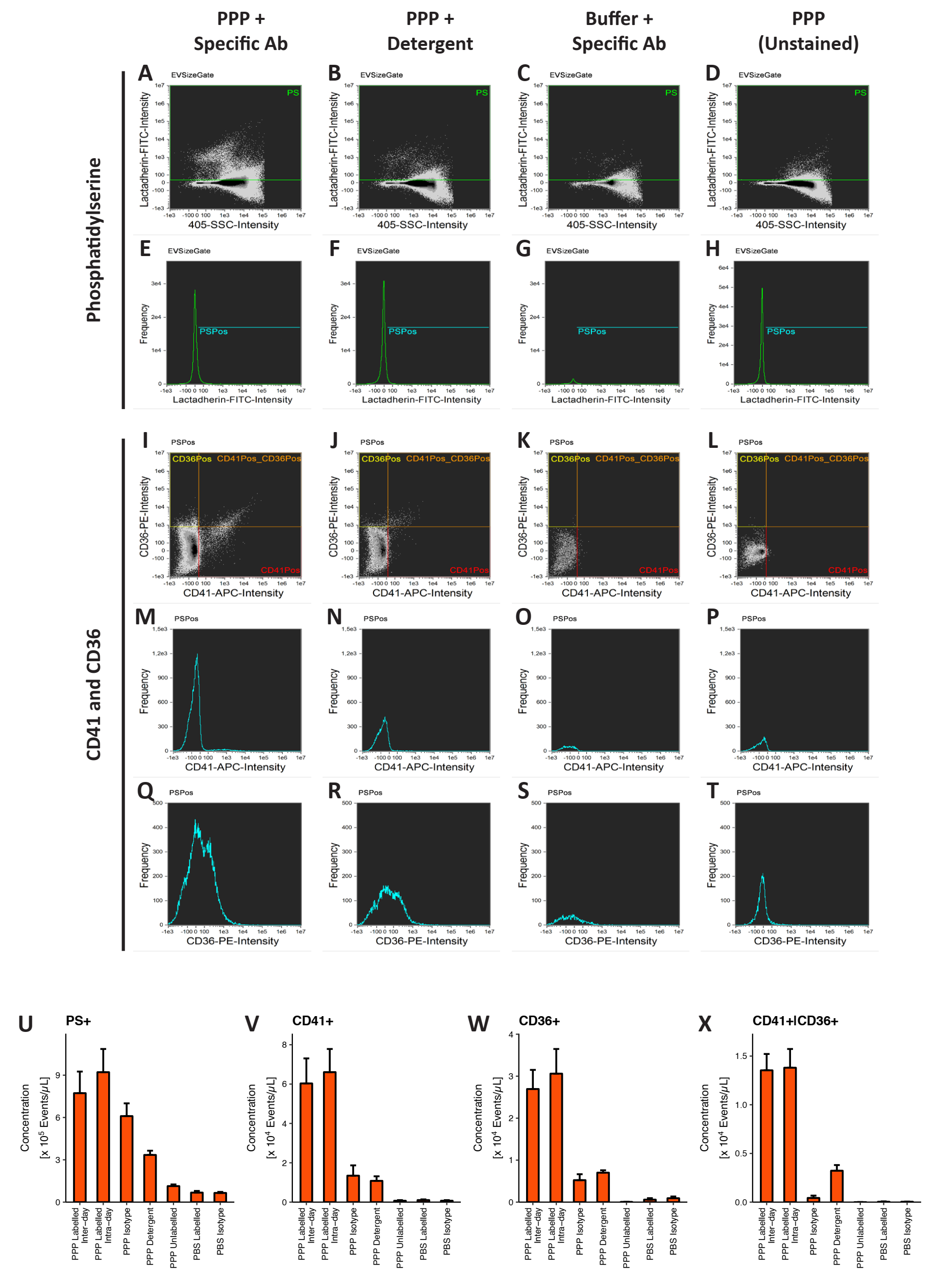


**Fig. S6: Controls for presence of EVs for data collected on ImageStream X Mk II. A-T)** Scatterplots and histograms depicting differences in signals in the **A-H)** phosphatidyserine-FITC and **I-T)** CD41-APC and CD36-PE channels between **A, E, I, M, Q)** PPP stained with specific antibody mixture, **B, F, J, N, R)** stained PPP after lysing with Triton X-100, **C, G, K, O, S)** buffer labelled with specific antibody, and **D, H, L, P, T)** unstained PPP. **U-X)** Depiction of concentrations differences of **U)** PS+, **V)** PS+|CD41+, **W)** PS+|CD36+, and **X)** PS+|CD41+|CD36+ events.


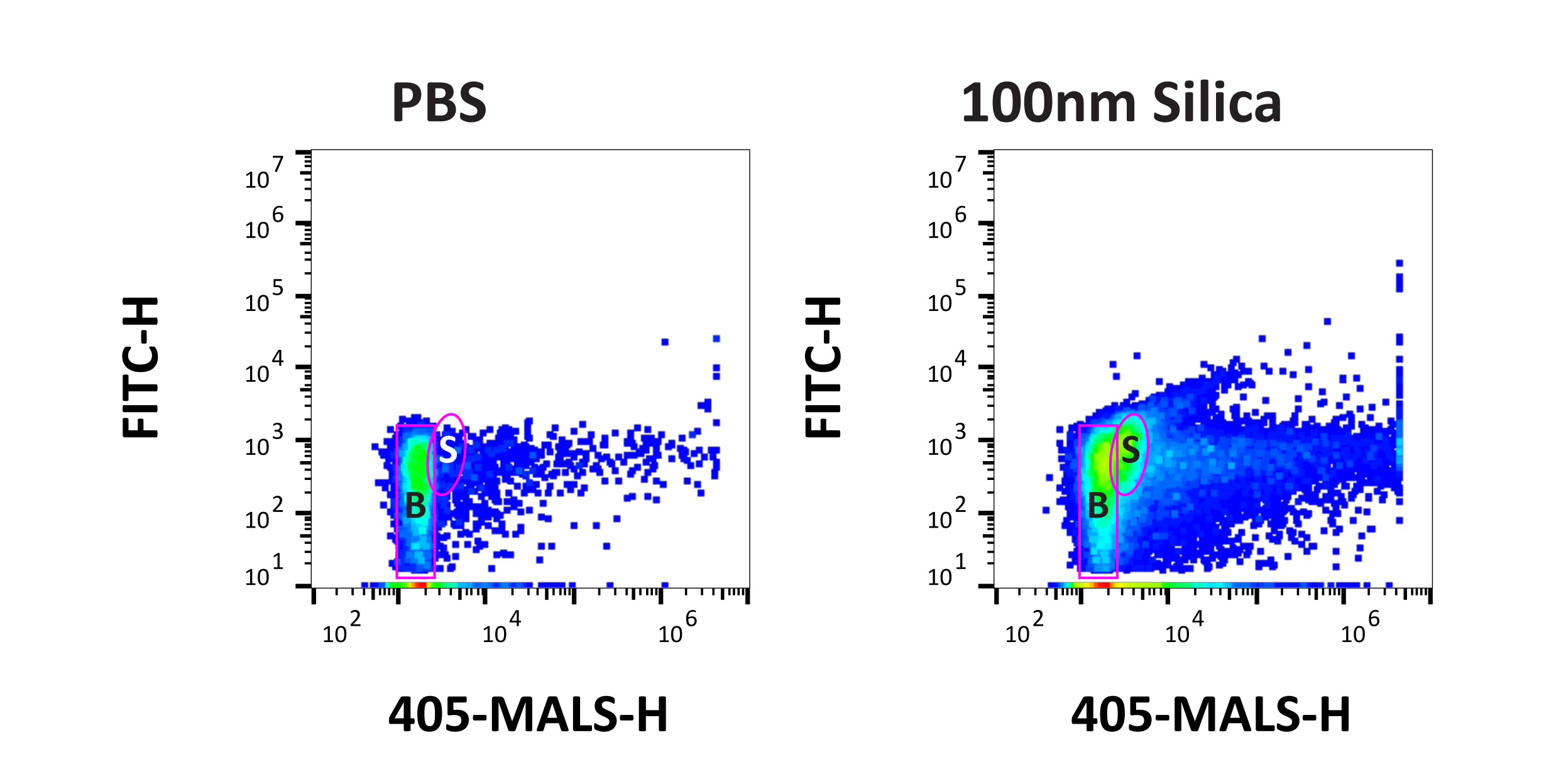


**Fig S7: Contribution of background to the concentration of 100nm dimly fluorescent silica nanospheres on the Apogee A60 Micro-PLUS.** The bulk of background is confined to the lower end of the MALS parameter and can be enclosed in a square gate (B) with only few events having higher values than the upper limit of this gate, as demonstrated in a buffer sample **(left panel)**. On the other hand, 100nm dimly fluorescent silica nanospheres **(right panel)** appear as a shoulder to this background population (S), which is not present in the buffer sample. Contribution of background to this population is minimal (100nm silica: 11750 ± 619 events/µL; PBS: 796 ± 619 events/µL).
